# Lactobacillus spp. for Gastrointestinal Health: Current and Future Perspectives

**DOI:** 10.3389/fimmu.2022.840245

**Published:** 2022-04-06

**Authors:** Elaine Dempsey, Sinéad C. Corr

**Affiliations:** ^1^ Trinity Biomedical Science Institute, School of Biochemistry and Immunology, Trinity College, Dublin, Ireland; ^2^ Department of Microbiology, Moyne Institute of Preventive Medicine, School of Genetics and Microbiology, Trinity College, Dublin, Ireland; ^3^ APC Microbiome Ireland, University College Cork, Cork, Ireland

**Keywords:** lactobacillus, probiotic, microbiota, gastrointestinal barrier, inflammation

## Abstract

In recent decades, probiotic bacteria have become increasingly popular as a result of mounting scientific evidence to indicate their beneficial role in modulating human health. Although there is strong evidence associating various Lactobacillus probiotics to various health benefits, further research is needed, in particular to determine the various mechanisms by which probiotics may exert these effects and indeed to gauge inter-individual value one can expect from consuming these products. One must take into consideration the differences in individual and combination strains, and conditions which create difficulty in making direct comparisons. The aim of this paper is to review the current understanding of the means by which Lactobacillus species stand to benefit our gastrointestinal health.

## Introduction

Ilya Ilyich Mechnikov (Elie Metchnikoff), a Nobel Laureate for his work on macrophage phagocytosis, is credited as the first to propose that the gut microbiota could be manipulated to benefit the host. Mechnikov believed that putrefactive activity of microbes in the intestine produced toxic substances that were damaging to the nervous and vascular systems and caused humans to age. He had observed that Bulgarian peasants consumed large quantities of yogurt and had a long life expectancy. He also observed that natural fermentation of food by lactic acid-producing bacteria prevented the growth of putrefactive organisms. In his book, titled ‘The Prolongation of Life’, he concludes that: “as lactic fermentation serves so well to arrest putrefaction in general, why should it not be used for the same purpose within the digestive tube?” (1). Although Mechnikov’s concept of aging by “intestinal auto-intoxication” has no scientific basis today, Mechnikov’s theories remain influential and have contributed to the commonly held opinion that Lactobacilli display important functional characteristics that contribute to gut health.

*Lactobacillus* is a genus of rod-shaped, gram-positive, non-spore-forming, facultative anaerobic bacteria of the phylum ‘Firmicutes’ (2, 3). *Lactobacilli* metabolise carbohydrates to produce lactic acid making them the largest genus within the lactic acid bacteria (LAB) group. As of March 2020 the 261 species of the *Lactobacillacae* were reclassified into 25 genera (including 23 novel genera) due to their extremely high genotypic, phenotypic and ecological diversity (4). For the purpose of this review, ‘*Lactobacillus’* will refer to those species previously classified as *Lactobacillus*. Traditionally, *Lactobacillus* species may be divided into three groups based on their metabolism. The obligate homofermentative group which ferment carbohydrates to produce lactic acid as the main by-product (e.g. *L. acidophilus* and *L. salivarius*), the facultatively heterofermentative group which, under certain conditions or with certain substrates, ferment carbohydrates to produce lactic acid, ethanol/acetic acid and carbon dioxide as by-products (e.g. *L. casei* and *L. plantarum*) and the obligately heterofermentative group which always ferment carbohydrates to produce lactic acid, ethanol/acetic acid and carbon dioxide as by-products (e.g. *L. reuteri* and *L. fermentum*) (5).

*Lactobacilli* have colonised multiple areas of the human body, most notably the digestive tract including the oral cavity, and the female genital tract (6). The association between *Lactobacilli* and humans is a mutualistic relationship, with *Lactobacillus* species offering the host aid in digestion of certain dietary substrates, as well as protection from pathogens, in return for accommodation and nutrients (7). Lactobacillus species possess qualities that are commercially desirable both as health supplements and as tools in the food technology sector. The main uses for *Lactobacilli* are in the manufacturing process of fermented dairy, meat, or vegetable foods and sourdough breads, and they are also widely used as probiotics i.e., live micro-organisms that, when administered in adequate amounts, confer a health benefit on the host (8, 9). *Lactobacilli* have been granted a ‘generally recognised as safe’ (GRAS) status from the U.S. Food and Drug Administration (USFDA) and ‘qualified presumption of safety’ (QPS) status from the European Food Safety Authority (EFSA) thus making their use in food manufacture relatively straightforward. Due to their economic importance, *Lactobacilli* are highly studied and, relative to other bacterial genus’, are well characterised in terms of genomics and also their interactions with humans in terms of both health and disease. These features make *Lactobacillus* species ideal probiotic candidates.

Considering the widespread media attention that the microbiota have attracted in recent years with many news outlets covering this link between microbes and health it is little wonder that the commercial probiotic market is worth approximately $54 billion USD worldwide (10). For a list including some of the most common *Lactobacillus* strains found in probiotic products and their sources see George Kerry et al. (11). Although the strain *L. rhamnosus* GG is one of the most heavily studied, *L. acidophilus* is the most commonly used in commercial products. For an in-depth review of common commercial *Lactobacillus* strains see the chapter by Tang and Zhao in the book ‘Lactic Acid Bacteria: Omics and Functional Evaluation’ (12).

In 2002 a joint Food and Agriculture Organisation (FAO) and WHO working group released guidelines for the evaluation of probiotics in food (8). The minimum requirements include: assessment of strain identity (genus, species, strain), *in vitro* tests to show probiotic effects (e.g. resistance to gastric acidity, digestive enzymes and bile acid, and anti-microbial activity against pathogens), safety assessment to prove that the probiotic product is safe for consumption and without contamination, and finally *in vivo* studies to authenticate the purported health claims of the product (13). In Europe, the EFSA considers the terms ‘probiotic’, ‘prebiotic’ and the words ‘live’ or ‘active’ when used in relation to bacteria, to be health claims. Legislation on products purporting to carry health claims are strictly controlled although in recent years countries including Spain, Denmark and the Netherlands have released national guidelines allowing use of the word probiotic under certain conditions. This has renewed appeals to the EU Commission to reconsider the strict regulation. Unfortunately, in the US and Canada the FAO/WHO guidelines are not followed and indeed the use of the term probiotic has not been controlled by legislation. This means that any product can use the word ‘probiotic’ on its packaging thereby making it extremely difficult for consumers to determine which products are genuine probiotics that may actually be beneficial for their health (14). 

In order to be considered efficacious, a probiotic must have the capacity to survive in the gastrointestinal (GI) tract, must resist the low pH of the stomach, must lack antibiotic resistance genes and must provide a clear benefit to the host (15). Of all probiotics, *Lactobacillus* species are the most widely used and studied (16). The main probiotic *Lactobacillus* species include: *L. acidophilus, L. brevis, L. casei, L. delbrueckii* subsp. *bulgaricus, L. delbrueckii* subsp. *lactis, L. fermentum, L. gasseri, L. helveticus, L. johnsonii, L. paracasei* subsp. *paracasei, L. plantarum, L. reuteri* and *L. rhamnosus*. There is much research into the potential health benefits of *Lactobacillus* species, although evidence indicates that many features of these probiotic bacteria are both species and strain -dependent (17). Despite this it has been observed that a single probiotic species may demonstrate improvement in different patient cohorts eg. *L. rhamnosus* GG (18) and additionally that a range of different probiotics or probiotic combinations may demonstrate efficacy in the same condition eg. *C. difficile* infection (19) highlighting the existence of conserved beneficial features. As is the case for many translational therapies, efficacy is not always maintained from *in vitro* observations through preclinical to clinical studies for a myriad of factors. Unfortunately, for many probiotics, one of these factors being that the mechanisms of action by which beneficial clinical outcomes are achieved have yet to be elucidated (20). The consequences for this mean that we are not utilising these tools to their full potential, opportunities for improving existing treatments may not be realised and we are at risk of probiotic treatments resulting in worse outcomes for particular subsets of patients (21). Additionally, mechanistic data may be required in order to gain approval from regulatory bodies for health claims – a mode of action is defined by the World Health Organisation (WHO) and EFSA as ‘a biologically plausible sequence of key events leading to an observed effect supported by robust experimental observations and mechanistic data’ (22). Kleerebezem and colleagues (23) propose the establishment of a translational pipeline connecting mechanistic insights to probiotic efficacy in order to improve the initial selection of probiotic strains by being able to predict their expected outcomes while supporting the design of the most appropriate clinical trials in well-defined subpopulations. They also suggest that this would be used in the inverse allowing us to predict explanations for observed clinical effects by drawing on existing knowledge of the probiotic modes of action. Determining the precise beneficial features of probiotics would certainly allow us to make better predictions for improved health outcomes.

On this note, further research is exploring ways to increase the efficiency, efficacy, safety and quality of probiotics by isolating probiotic-derived biomolecules. These have been described as postbiotics, paraprobiotics, heat-killed probiotics, Tyndallised probiotics among others: generally referring to metabolic products or secreted products of the bacteria, non-viable microbial cells (intact or broken) or crude cell extracts; specifically this includes enzymes, secreted peptides/proteins, bacteriocins, short chain fatty acids (SCFA), organic acids and cell envelope components of bacteria including peptidoglycans, teichoic acids, cell surface proteins and cell wall polysaccharides (24). The International Scientific Association for Probiotics and Prebiotics (ISAPP) has released a consensus statement on the definition of postbiotics establishing it as a “preparation of inanimate micro-organisms and/or their components that confers a health benefit on the host. Effective postbiotics must contain inactivated microbial cells or cell components, with or without metabolites, that contribute to observed health benefits”. (25). Postbiotics maintain several advantages over probiotics as described by Pique et al. (26): (I) No risk of translocation from the gut lumen to blood among vulnerable subjects, (II) No risk of acquisition and transfer of antibiotic resistance genes, (III) No risk of interference with normal gut colonisation in neonates, (IV) Release of active molecules from the disrupted inactivated cells, pass through the mucus layers and stimulate epithelial cells more directly, (V) Loss of viability by cell lysis can produce further more complex beneficial effects and (VI) Easier to extract, standardize, transport, and store. Accordingly, the use of postbiotics may very well represent a much-improved alternative to live probiotics and would be a likely replacement for them in future. A recent review has nicely summarised the composition and beneficial functions of postbiotics from *Lactobacillus* species (27). In short, postbiotics derived from *Lactobacillus* comprise a range of molecules which have various beneficial effects including immunomodulation, epithelial barrier protection, anti-pathogenic effects and anti-tumour effects.

*Lactobacilli* have demonstrated efficacy in treating various conditions including bacterial vaginosis, atopic dermatitis, and upper respiratory tract infections (28–30). However, as first proposed by Mechnikov over 100 years ago, the majority of *Lactobacillus* probiotics are consumed with a view to improving GI health. In the century since this hypothesis, interest and knowledge surrounding this subject has grown massively, however the potential for further growth in this area is exponential and much more work will be required before we fully understand and profit from the complexities of the relationships between *Lactobacillus* and gut health.

## *Lactobacillus* spp. and Intestinal Barrier Integrity

The GI mucosa is the largest and one of the most critical barrier sites of the body where foreign antigens, microbes and potential pathogens come into close contact with the host’s immune system. It is a semi-permeable barrier which allows for the absorption of nutrients and immune sensing while restricting the influx of potentially harmful antigens or microbes. The GI barrier is composed of four major elements: the commensal microbiota, the mucus layer – which contains secretory IgA molecules (sIgA) and anti-microbial peptides, the intestinal epithelial cell (IEC) monolayer, and the gut associated lymphoid tissue (GALT) - which constitutes various populations of immune cells in compartments along the GI tract. The complexity of regulating this semi-permeable barrier is mitigated by dynamic inter-regulation between these elements which work together to maintain intestinal barrier integrity and homeostasis (31). Loss of intestinal barrier function has been implicated as an early event in the pathogenesis of various GI disorders, such as coeliac disease and inflammatory bowel disease, as well as systemic disorders including type I diabetes, obesity and multiple sclerosis (31).

**Figure 1:**
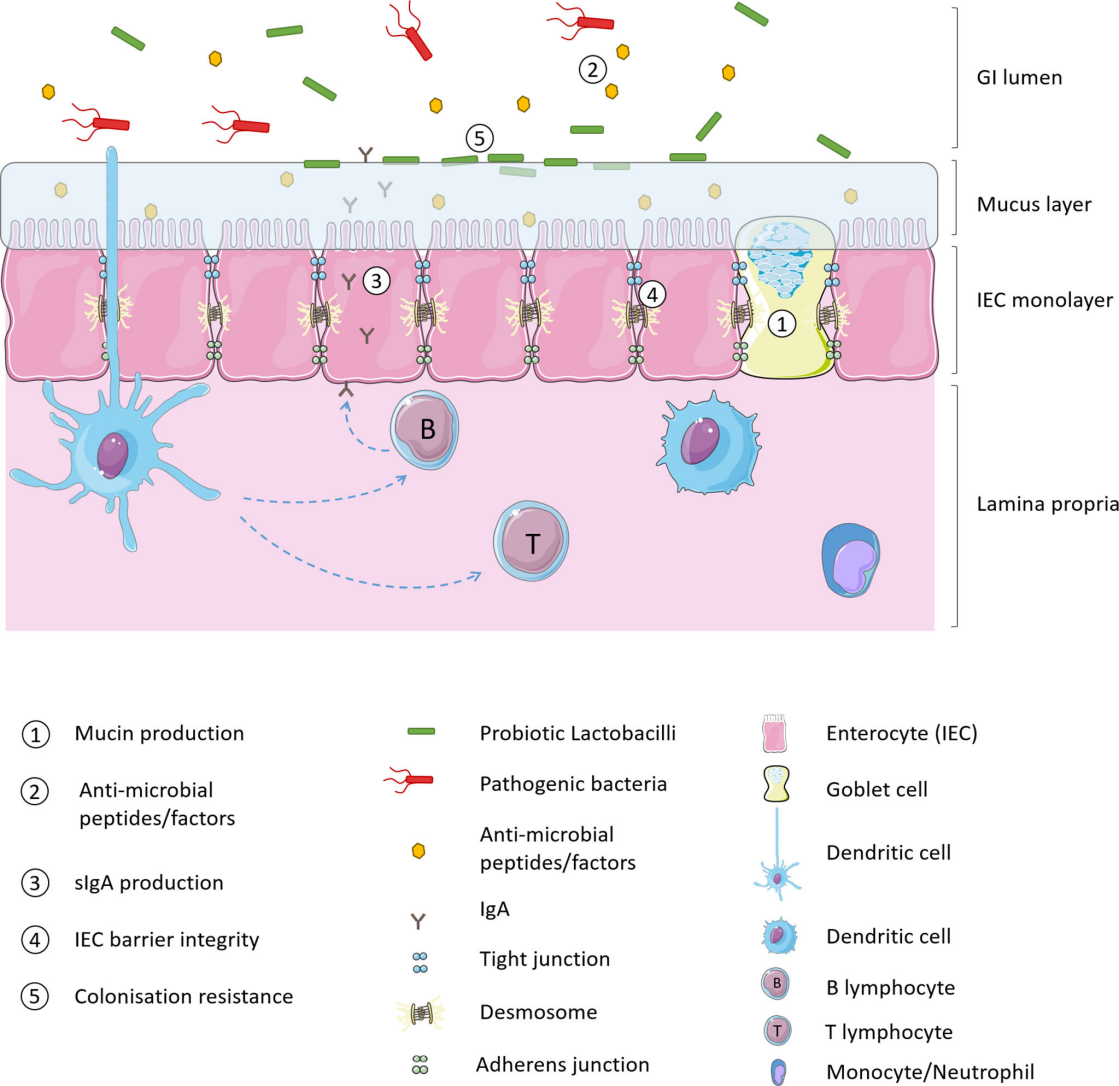
Probiotic mechanisms of intestinal barrier enhancement.

Intestinal barrier function may be enhanced with the intake of non-pathogenic micro-organisms which augment the physical barrier of the mucus layer, enhance innate defence against pathogens and decrease paracellular permeability of IECs (32). *Lactobacillus* strains consumed as probiotics are thought to modulate the native intestinal microbiota and improve health *via* multiple mechanisms of action. As illustrated in **Figure 1**, probiotics strengthen intestinal barrier function by increasing mucus production, stimulating release of anti-microbial peptides, and production of secretory immunoglobulin A (sIgA) production, increasing tight junction integrity of IECs and providing a competitive resistance against pathogens such as for host colonisation receptors (33, 34).

### Mucus Production

Goblet cells (GC) of the GI tract express rod-shaped mucins which either adhere to the epithelium or are released into the GI lumen. These mucins are highly glycosylated and link together *via* di-sulfide bonds to form a glycoprotein matrix that shields the intestinal epithelium from gut luminal contents (containing digestive enzymes), prevents interaction between pathogenic antigens/bacteria and the epithelial monolayer, and also aids GI motility. The mucus layer is generally between 50-800 µm thick and in healthy individuals the first 30 µm closest to the epithelial surface should be free of microbes. *Lactobacillus* species are believed to enhance intestinal barrier defence by promoting mucus secretion. *In vitro* studies have demonstrated that conditioned media from *L. casei* T21 can up-regulate the mucosal protective *MUC2* gene in colonic epithelial cells (Caco2 and HT29) challenged with *C. difficile* (35). Although it has been proposed that acid may stimulate enteric cells to produce mucins (36) incubating HT29 cells with lactic acid did not replicate these findings indicating that other substances secreted by *L. casei* T21 are responsible for the increased gene expression. Similar results have also been obtained in the Caco-2 intestinal epithelial cell line using *L. casei* GG (37). In terms of *in vivo* studies, *L. rhamnosus* CNCM I-3690 has recently been shown to protect and/or restore the GC population and protect mucus layer thickness in mice following low-grade colon inflammation (38). Similarly, mice administered one of two strains of *L. reuteri* (*L. reuteri* R2LC or 4659) and exposed to DSS colitis displayed reduced colitis severity which the authors attribute at least partly to the increase in mucus thickness seen in mice given the probiotic both in control and inflammatory conditions (39).

The commercially available probiotic VSL#3 contains a combination of eight lactic acid producing bacteria of which four are *Lactobacilli* (*L. plantarum, L. delbrueckii* subsp. *Bulgaricus*, *L. casei*, *L. acidophilus*, *Bifidobacterium breve*, *B. longum*, *B. infantis* and *Streptococcus salivarius* subsp. *thermophilus*). Although the contribution of each bacterial strain cannot be clarified, both *in vitro* and *in vivo* experiments by Caballero-Franco et al. (40) using this probiotic in rats have indicated enhancement of the mucus layer measured by over-expression of mucin genes and increased basal luminal mucin content. Conversely, a similar study in mice failed to show altered mucin expression or mucus layer thickness using this probiotic (41). Further work is required to determine whether the *in vitro* effects of probiotics on mucus production are maintained *in vivo*.

### Anti-Microbial Peptides/Factors

Host-produced GI anti-microbial peptides are generally categorised into cathelicidins and defensins. Cathelicidins are α-helical cationic peptides constitutively expressed in the GI tract which may also be activated by butyrate. Butyrate is produced by the enteric microbiota however few studies have examined the effect of probiotics on cathelicidin expression. Defensins are small, cationic peptides further classified into β-defensins, produced by epithelial cells throughout the intestine, and α-defensins, expressed in the small intestine. Defensins are constitutively expressed in the GI tract and display anti-microbial activity against many bacteria, fungi and some viruses. *L. acidophilus* PZ1138 and *L. fermentum* PZ1162, were shown to induce expression of human β-defensin-2 gene in Caco-2 cells *via* classic pro-inflammatory mechanisms (42). *L. reuteri* (FINELACT^®^) administered to broiler chicks was associated with anti-microbial peptide modulation in the cecum and ileum in addition to upregulation of pro-inflammatory mediators (43).

In addition to host-derived anti-microbial peptide stimulation, commensal bacteria also produce anti-microbial factors to aid in host barrier defence. These factors include short chain fatty acids (SCFA), hydrogen peroxide and bacteriocins. *Lactobacilli* alter luminal pH by producing lactic acid. This inhibits the growth of some bacteria and damages the outer cell membrane of Gram-negative bacteria, including *E. coli* O157:H7, *Pseudomonas aeruginosa*, and *Salmonella enterica* serovar Typhimurium making them more vulnerable to other anti-microbial molecules (44). Anti-microbial activity by *L. johnsonii* NCC533 has been associated with lactic acid and hydrogen peroxide production (45). Bacteriocins are small, ribosomally synthesised, heat-stable peptides produced by many species of bacteria which function to inhibit the growth of (bacteriostatic), or kill (bactericidal), other bacteria (46). Bacteriocins produced by Gram-positive bacteria generally exert their antibiotic effects by destabilisation of membrane function, typically against other Gram-positive bacteria, though some Gram-negative bacteria may also be susceptible (47). *Lactobacillus* strains produce SCFAs including acetate, propionate and butyrate, which have been shown to shown to increase transepithelial electrical resistance and stimulate the formation of tight junction in Caco-2 intestinal epithelial cells *in vitro via* inhibition of the NLRP3 inflammasome and autophagy (48). *L. plantarum* strains produce several bacteriocins which demonstrate anti-microbial activity against food borne pathogens such as *Listeria monocytogenes* as well as food spoilage bacteria are applied in food production to reduce the use of chemical preservatives (49). Corr *et al.* (50) demonstrated that Abp118 produced by *L. salivarius* UCC118 *in vivo* protects mice against *L. monocytogenes* infection. Two other bacteriocins analogous to Abp118 have since been identified by comparative genome hybridisation analysis from *L. salivarius* DPC6488: salivaricin L and T. Both bacteriocins demonstrated inhibitory activity towards *L. delbrueckii* subsp *bulgaricus* LMG 6901 with salivaricin L additionally inhibiting *L. monocytogenes* NCTC 11994 and *L. innocua* DPC3572 (51). 

### Secretory IgA

The production of IgA is an important strategy utilised by the GI tract to generate immune protection in a non-inflammatory mode (52). IgA dimers (secreted by intestinal B cells located in Peyer’s patches or lamina propria) interact with the polymeric IG receptor (pIgR) on the basolateral surface of epithelial cells, translocate to the surface of the epithelial cells and are released as sIgA (53). sIgA primarily promotes the maintenance of suitable commensal bacterial communities in the gut by binding dietary antigens and potential pathogens in the mucus and down-regulating the expression of pro-inflammatory bacterial epitopes on commensal bacteria (54). Furthermore, sIgA enhances the intestinal barrier by blocking microbial components involved in epithelial adherence, facilitating intraepithelial defence against pathogens and microbial products and enabling antigen sampling (55). In addition, locally released IgA dimers function to remove micro-organisms that have breached the epithelial barrier by facilitating their removal or promoting their clearance by binding to the CD89 receptor on immune cells such as dendritic cells, neutrophils and other phagocytes (56). Although commensal bacteria are believed to induce sIgA expression in the GI tract the mechanisms are not well understood, although there appear to be differences in the microbes responsible for small intestine and large intestine sIgA induction (57). Various *Lactobacillus* strains including *L. paracasei* MCC1849*, L. gasseri* SBT2055, and *L. plantarum* AYA are known to increase sIgA levels in the small intestine (58–60). In a clinical trial of children 12 to 24 months old, supplementation with *L. plantarum* IS-10506 increased sIgA faecal titres and a significant positive correlation was observed between this and TGF-β1/TNF-α ratios (61). The authors propose a probiotic induced immune activation of TGF-β1, which in turn increases the production of sIgA.

### Epithelial Cell Barrier

As previously described, IECs form a monolayer of cells which act as a physical barrier between the external environment of the gut lumen and the host’s immune system. The integrity of this barrier is ensured by tight junctions (TJ) which are multi-protein complexes that bind the cells tightly together as well as adherens junctions, gap junctions and desmosomes. TJs are located towards the apical side of the epithelial cells. They consist of transmembrane proteins (claudin, occludin, and junctional adhesion molecules) which interact extra-cellularly with similar proteins of TJs in neighbouring cells and intra-cellularly with the cells own cytoskeleton *via* zonula occludens (ZO) proteins and filamentous actin (62). Loss of TJ integrity has been observed in chronic inflammatory disease, and mechanisms of disrupting TJ proteins in order to breach the GI barrier have been observed in infection by enteric pathogens such as *C. difficile*, *E. coli, Salmonella* Typhimurium*, C. rodentium, Vibrio cholera* among others (62). It has been demonstrated that *L. rhamnosus* GG ATCC 53103 up-regulates ZO-1, claudin and occludin expression in Caco-2 cells (63). This probiotic strain has been observed to increase levels of ZO-1 expression and enhance distribution of claudin-1 protein as a protective mechanism against enterohemorrhagic *E. coli* O157:H7 infection (64). Increased expression of ZO and occludin was also observed using various *L. plantarum* strains (*L. plantarum* WCSF1, CGMCC 1258, and MB 452) (65–67). *L. plantarum* WCSF1 administration into the duodenum of healthy human subjects increased ZO-1 and occludin staining in the vicinity of TJ structures *via* activation of TLR-2 (65). The addition of a TLR-2 agonist PCSK to Caco2 monolayers *in vitro* increased staining of occludin in TJ regions and was protective against epithelial barrier disruption. TLR-2 ligand binding leads to PKC activation which has been demonstrated to cause translocation of tight junction components (68) thereby it is likely that barrier integrity is enhanced by alterations to composition of tight junction proteins rather than an increase in these proteins. *Lactobacillus* species may also stabilise adherens junctions by increasing expression of E-cadherin, as well as by strengthening the E-cadherin/β-catenin complex (which connects adherens junctions to the cytoskeleton) *via* enhanced phosphorylation of β-catenin (69). In a clinical study of small intestine barrier function, biopsy samples demonstrated that *L. plantarum* strain TIFN101 and to a lesser extent *L. plantarum* WCFS1 and CIP104448, modulated an increase in gene expression of TJ and adherens junction proteins (70).

### Competitive Resistance 

**Figure 2:**
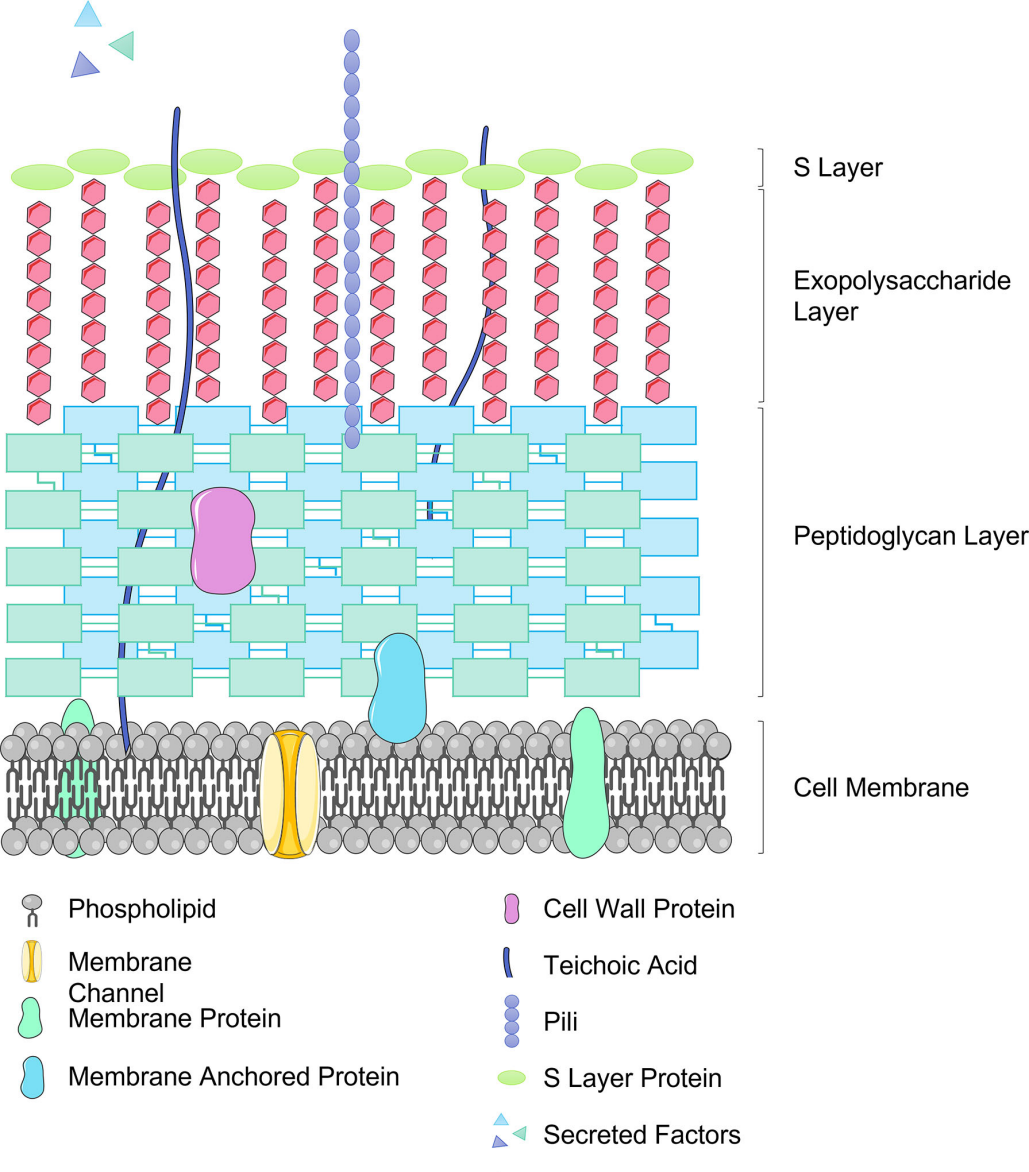
Representation of the Lactobacillus cell surface structure including important effector molecules.

*Lactobacilli* also aid intestinal barrier resistance to invading pathogens by competing for binding sites on IECs, glycoproteins in the mucus layer or to the plasminogen of extracellular matrix (71). In order to facilitate the necessary interactions with host cells, *Lactobacillus* species display various different components on their outer surface. These may include cell wall proteins, S-layer proteins, pili proteins, and moonlight proteins (72) (see **Figure 2**). These surface proteins facilitate adhesion of *Lactobacilli* to the host, for example LPXTG proteins found in several *Lactobacillus* strains are cell surface proteins covalently bound to the peptidoglycan layer and can bind to both mucus and epithelial cells (73). Several *Lactobacillus* strains possess a crystalline, glycoprotein surface layer, also known as the S-layer, non-covalently anchored to the peptidoglycan cell wall (74). The S-layer S-proteins of *L. acidophilus* ATCC 4356 have demonstrated anti-viral activity against alphavirus and flavivirus infection of 3T3 cells by blocking pathogen adhesion to C-type Leptin receptors (DC-SIGN) an attachment factor which strongly promoted viral infection (75). Further work is required to elucidate the mechanism for this, which may be multi-faceted, though the time-dependant aspect of the anti-viral function may indicate that S-layer proteins are activating downstream anti-viral signalling pathways.

Pili are long protein structures, first observed in a non-pathogenic bacteria in *L. rhamnosus* GG, which protrude from the bacterial cell playing a major role in adhesion to the epithelium. In *L. rhamnosus* GG (ATCC 53103) SpaC pili have been demonstrated to out-compete the pathogenic *Enterococcus faecium* (76).

Moonlighting proteins are multifunctional proteins in which one polypeptide chain performs more than one unrelated biochemical or biophysical function (77). In *Lactobacilli*, moonlighting proteins may have a primary function as intracellular proteins but are also found on the cell surface where they facilitate adhesion, for example, *L. plantarum* 299v (78), *L. acidophilus* (79), *L. reuteri* ZJ617 (80), display GAPDH on their surface to mediate adhesion and colonisation of the GI tract. So far in the case of *L. plantarum* 299v it has been demonstrated that this results in competitive exclusion and displacement of pathogenic bacteria (81). The mechanism for the secretion of moonlighting proteins to the cell surface has not yet been elucidated.

*L. rhamnosus* R0011 and *L. acidophilus* R0052 adhere to Hep-2 and T84 intestinal cell lines *in vitro* preventing the binding of enterohemorrhagic *E. coli* and enteropathogenic *E. coli* (82). In Caco-2 cells, various strains of *L. reuteri* (LR5, LR6, LR9, LR11, LR19, LR20, LR26, and LR34) have been shown to adhere and inhibit and displace the binding of *E. coli* ATCC 25922, *S.* Typhi NCDC 113, *L. monocytogenes* ATCC 53135, and *E. faecalis* NCDC115 (83). It should be noted that competition for binding sites is species and strain -specific; *L. rhamnosus* ATCC 53103, *L. gasseri* DSM 20243*, L. casei* ATCC 393 and *L. plantarum* ATCC 14917 pre-treatments did not block enterohemorrhagic *E. coli* binding to human colon epithelial cell line C2BBe1 cells (although the *L. rhamnosus* strain prevented internalisation of *E. coli* into the cell line) (84). In a chronic stress model *in vivo*, pre-treatment with *L. helveticus* R0052 and *L. rhamnosus* R0011 reduced commensal adherence and translocation (85). Interestingly, in a hemorrhagic shock model *in vivo, L. rhamnosus* LMG P-22799 but not *L. fermentum* NumRes2 reduced bacterial translocation and cytoskeleton rearrangement despite both strains displaying similar pathogen exclusion properties *in vitro* in Caco2 cells (86). Indeed, *L. fermentum* NumRes2 increased bacterial translocation, primarily *Lactobacillus* spp., to the spleen highlighting the need for careful characterisation of the effects of individual.

## *Lactobacillus* spp. and Gastrointestinal Infection

Understandably, the beneficial impact on gut health is one of the most widely studied topics in probiotic research. As discussed in the previous section, *Lactobacilli* protect the intestinal barrier from infection by promoting mucus production and barrier-related proteins, secreting anti-microbial substances such as SCFAs, bacteriocins and hydrogen peroxide which inhibit the growth of or kill pathogens, by modulating the host’s immune response to pathogens, and preventing adherence of pathogens and competing for binding sites. Thus, *Lactobacilli* are capable of preventing intestinal damage caused by certain bacterial infections. *Lactobacillus* probiotics have been demonstrated to inhibit the development of infection by pathogenic bacteria, such as *C. difficile* and *C. perfringens* (87), *Campylobacter jejuni* (88), *S.* Enteritidis (89)*, E. coli* (90), *Staphylococcus aureus* (91), and *Yersinia* (92), among others. Two major GI disorders resulting from infection, *H. pylori* infection and antibiotic-associated diarrhoea, have been shown to greatly benefit from *Lactobacillus* probiotics and are outlined below.

### *H. pylori* Infection and Lactobacilli

*H. pylori* infection is one of the most common bacterial infections in the world with more than half of the global population infected; though prevalence ranges from 24% in Oceania to 70% in Africa (93). *H. pylori* infects the epithelial lining of the stomach causing disorders such as peptic ulcer disease, chronic gastritis, and gastric cancer although many infected individuals are asymptomatic (94). Twenty percent of infected patients develop symptomatic gastritis, gastric or duodenal ulcers, gastric adenocarcinoma, or non-Hodgkin’s gastric lymphoma. The current recommended treatment for *H. pylori* infection involves multiple antibiotic drugs as well as a proton pump inhibitor however the effectiveness of this treatment is decreasing as *H. pylori* antibiotic resistance rises. The addition of a *Lactobacillus* probiotic (*L. casei* DN-114 001 (OAC-LC) and *L. casei* Shirota separately) and an *L. acidophilus* LB postbiotic have been shown to improve the efficacy of this therapy in various randomised controlled trials (95–97), however some trials have found no or only slight beneficial effects (98–101). Although the probiotic *L. johnsonii* NCC533 failed to eradicate *H. pylori* infection when administered alone, it did decrease inflammatory scores and urea breath test (used for the diagnosis of *H. pylori* infection) values (102, 103).

Cell-free spent culture supernatants (CFCS) derived from *L. casei* Shirota exhibited pH-dependant bactericidal activity against *H. pylori in vitro* (104). The CFCS of *L. johnsonii* NCC533 and *L. acidophilus* LB both resulted in the loss of *H. pylori* viability (105–107). Furthermore, the CFCS from these three *Lactobacillus* strains resulted in altered morphology of *H. pylori* bacteria to U-shaped or coccoid forms which are dormant forms of the bacteria with the coccoid form being less capable of colonising and inducing inflammation (108, 109). *L. johnsonii* NCC 533 and *L. casei* Shirota are also known to produce bacteriocins which are active against *H. pylori* (110). *H. pylori* is a spiral-shaped bacterium with multiple flagella allowing it to swim in the gastric mucus layer and interact with epithelial cells, an ability which is required for colonisation in the stomach (111). *L. casei* Shirota has been demonstrated to cause *H. pylori* to lose its flagellar motility due to transformation into dormant forms with no flagella and also by secretion of small anti-microbial compounds which inhibit swimming ability (104). Similarly, *L. johnsonii* NCC 533 also secretes compounds that inhibit the swimming ability of *H. pylori* (112). In order to survive in the low pH of the stomach, *H. pylori* expresses urease as a surface protein to neutralise the surrounding acidic environment. CFCSs from *L. acidophilus* LB and *L. johnsonii* La1 have been demonstrated to reduce urease activity of *H. pylori* (105, 106). In terms of adherence, *L. acidophilus* CFCS prevented the adhesion of *H. pylori* onto human HT-29 cells resulting in the death of adhering cells and reducing the urease activity of remaining adherent cells causing their lysis (105).

### Antibiotic-Associated Diarrhoea and Lactobacilli

Antibiotic-associated diarrhoea (AAD) results from disruption of the normal microbiota of the gut by antibiotics with symptoms ranging from mild diarrhoea to more serious disease like pseudomembranous colitis (PMC) (113). AAD occurs in 5-30% of patients receiving antibiotics either during antibiotic therapy or up to 2 months after cessation of treatment. One of the major pathogens associated with AAD is *C. difficile*, responsible for 10-30% of normal AAD cases and 90-100% of severe cases such as PMC (114). Although other microbes including *C. perfringens, S. aureus* and *Klebsiella oxytoca* are associated with this disorder, they are not common (113). As the cause for AAD is known to be disruption of the normal intestinal microflora, and also due to the fears surrounding anti-microbial resistance, recent therapeutic research has focused on the use of probiotics or faecal microbiota transplantation to restore microbial equilibrium (115, 116). Though the mechanism of action of probiotics is not explicitly known in this case their efficacy seems to be maintenance of gut flora, out-competing pathogenic bacteria, preservation of intestinal barrier function and potentially immunomodulation. Treatment with several *Lactobacillus* strains including *L. rhamnosus* GG (ATCC 53103) and *L. gasseri* have been shown to be effective as a preventive measure for AAD (117). However, the effects are strain-dependent. A systematic review examined 51 randomised controlled trials and found that *L. rhamnosus* GG was significantly more effective than other probiotics, however *L. casei* species were most effective against *C. difficile* infection (118). Another recent review demonstrated similar results in children concluding that *L. rhamnosus* GG (ATCC 53103) can be safely given to prevent AAD and additionally to manage symptoms of acute gastroenteritis (119).

## *Lactobacillus* spp. and Intestinal Inflammation

In humans, the immune system can be divided into the innate immune system and the adaptive immune system. Innate immunity is the first line of immune defence and is a non-specific response which acts as an immediate reaction to pathogens. Phagocytic cells such as natural killer (NK) cells, macrophages, monocytes and neutrophils recognise pathogenic targets and engulf and destroy them. Antigen presenting cells (APC) such as dendritic cells (DC) maybe activated *via* the innate response and in turn activate the adaptive immune response. The adaptive immune response relies largely on activation and differentiation of B and T cells. B cells recognise antigens *via* B cell receptors and act by secreting antibodies (humoral immunity). T cells recognise antigens *via* T cell receptors and differentiate into T helper cells (Th; CD4+) or cytotoxic T cells (CD8+). Th cells recognise antigen *via* MHC class I complexes and CD8+ cells do this *via* MHC class II complexes. Th cells differentiate into Th1 or Th2 effector cells which activate and regulate macrophages (Th1) and B cells (Th2) while CD8+ cells convert into cytotoxic T cells. In the GI tract the immune system is made up of the epithelial layer, the lamina propria and the gut associated lymphoid tissue. The GALT is populated by B and T cells as well as plasma cells, macrophages and M cells. APCs in Peyer’s patches take IgA antigen from epithelial cells to activate T cells and also transport it to lymphoid tissue of the lamina propria and mesenteric lymph nodes. M cells present in Peyer’s patches of the small intestine transport antigens, macromolecules, micro-organisms and inert peptides from the gut lumen into the tissue *via* adsorptive endocytosis. These antigens may then activate the innate and adaptive immune systems.

As alluded to in the previous sections, *Lactobacilli* play an immunological role within the GI tract of the host, strengthening the intestinal barrier and conferring protection from potential pathogens. *Lactobacilli* can interact with both the innate and adaptive immune response systems *via* micro-organism-associated molecular patterns (MAMPs) interacting with pattern recognition receptors such as Toll-like receptors (TLRs), nucleotide-binding oligomerization domain (NOD) receptors and C-type lectins expressed on immune cells or on tissues including intestinal epithelium (120). The *Lactobacillus* cell envelope comprises several types of molecules which act as MAMPs including the peptidoglycan multi-layer, teichoic acids (lipoteichoic acid (LTA) bound to the cell membrane and wall teichoic acid bound to the peptidoglycan layer), exopolysaccharides (EPS) along with cell surface adhesion molecules previously discussed (see **Figure 2**). The immunomodulatory effect of *Lactobacilli* is achieved with the release of cytokines, including interleukins (IL), tumour necrosis factors (TNF), interferons (IFN), transforming growth factor (TGF), and chemokines from immune cells (121). The inflammatory process depends on pro-inflammatory versus anti-inflammatory cytokines and in this way probiotics may act in an immunoregulatory or immunostimulatory manner. Immunoregulatory probiotics decrease inflammatory responses protecting the host against autoimmune diseases, inflammatory bowel disease and allergy and are characterised by IL-10 and regulatory T cell (Treg) production. IL-10 is an anti-inflammatory cytokine produced by monocytes, T cells, B cells, macrophages, NK cells and DCs to inhibit pro-inflammatory cytokines, chemokines and chemokine receptors protecting against intestinal inflammation. Immunostimulatory probiotics defend the host against infection and cancer development activating NK cells and developing Th1 cells *via* IL-12 production, and also defend the host against allergy by balancing Th1 and Th2 production. Mounting evidence would suggest that probiotic *Lactobacilli* have the potential to prevent or treat certain inflammatory conditions (122).

The activation of specific immune receptors by MAMPs on Lactobacillus species has been characterized to an extent. Peptidoglycan of *L. casei* Shirota, *L. johnsonii* JCM 2012 and *L. plantarum* ATCC 14917 has been shown to down-regulate IL-12 production *via* TLR2 (123). Peptidoglycan from *L. rhamnosus* CRL1505 demonstrated an enhancement of innate and adaptive immune responses ameliorating the Th2 response when administered nasally in mice (124). LTA of *L. plantarum* has been shown to elicit an anti-inflammatory response in both human and porcine intestinal epithelial cells *via* inhibition of IL-8 (125, 126). The knockout mutant for the SpaCBA pilus of *L. rhamnosus* GG demonstrated that not only are these pili essential for adhesion but also the knockout demonstrated an increase in IL-8 likely *via* LTA TLR2 signalling which suggests an immunomodulatory role for this adhesion molecule (127). The protective exopolysaccharide layer has also demonstrated immunomodulatory capabilities with EPS from *L. rhamnosus* RW-9595M inducing macrophage production of IL-10 and no induction of TNF-α, IL-6, or IL-12 (128) and *L. plantarum* 14 EPS decreasing the IL-6 and IL-8 production in response to an enterotoxigenic *E. coli* challenge in porcine epithelial cells (129). In mice, EPS derived from *L. delbrueckii subsp.bulgaricus* OLL1073R-1 fermented yogurt had an immunostimulatory effect, activating natural killer (NK) cells and inducing IFN-γ production in the spleen (130).

Some immunomodulatory effects are mediated by the metabolites of *Lactobacillus*, such as SCFAs, in particular, propionate, acetate, and butyrate. These postbiotics bind to specific receptors on intestinal epithelial cells to inhibit pro-inflammatory activity and Treg suppressive effects of neutrophils and macrophages (131–133). Indeed butyrate enemas have demonstrated efficacy and become an accepted treatment for diversion colitis though this is believed to be due to a relaxation effect on smooth muscle (134).*Lactobacilli* are also capable of producing antioxidants like glutathione (GSH) and can induce reductions in oxidative stress. Two strains of *L. bulgaricus* (*L. delbrueckii* subsp. *bulgaricus* B3 and A13) have been demonstrated to reduce lipid peroxidation, increase measurements of antioxidant enzymes, and reduce oxidative stress in a rat model of colitis (135). In a mouse model of gastric damage *L. fermentum* Suo significantly reduced malondialdehyde (MDA; a measure of oxidative damage) concentrations and serum concentrations of IL-6, IL-12, TNF-α, and IFN-γ (136). *L. casei* 114001 administered to rats increased the antioxidant capacity of plasma, liver and intestines and decreased MDA plasma concentration (137). In healthy human subjects, *L. casei* capsules administered with prebiotic inulin significantly decreased MDA and glutathione disulphide (GSSG; another measure of oxidation) concentrations and increased concentrations of antioxidant indicators: GSH, total GSH (GSHt) and free sulfhydryl group (-SH) in the plasma (138). Pre-treatment with *L. acidophilus* NCDC15 with inulin and *L. rhamnosus* GG MTCC 1408 with inulin in a model of colon cancer in mice lead to a reduction in MDA and an increase in antioxidants GSH-reductase, GSH-peroxidase and superoxide dismutase as well as fewer dysplastic changes (139).

*Lactobacilli* may also modulate the immune system by secretion of proteinaceous compounds. Proteins p40 and p75 released from *L. rhamnosus* GG ATCC 53103 both activated the Akt signalling pathway, inhibiting TNF-a –induced apoptosis in human and murine colonic epithelial cells and murine colon explants (140). Pre-treatment with *L. rhamnosus* GG milk prior to induction of dextran sulphate sodium –induced colitis in mice significantly reduced colonic inflammation and injury, suppressing cytokine-induced apoptosis and reducing H_2_O_2_-induced disruption of TJs. Depletion of two soluble proteins found in *L. rhamnosus* milk, p40 and p75, abolished these anti-inflammatory effects (141). *L. rhamnosus* GG ATCC 53103 increased production of the heat-shock proteins HSP25 and HSP72 in murine colon cells *via* secretion of soluble peptides which function *via* activation of MAPK signal transduction pathway (142).

There have been many reports of *Lactobacilli* influencing the immune system while also enhancing the intestinal barrier. *In vitro, L. acidophilus* PZ1138, *L. fermentum* PZ1162, and *L. paracasei* LMG P-17806 induced expression of human β-defensin-2 gene in Caco-2 cells *via* modulation of nuclear factor kB (NF-kB) and the activator protein 1 (AP-1) resulting in IL-8 expression (42). *L. salivarius* Ls33 peptidoglycan induced anti-inflammatory IL-10 production, and stimulated Treg responses *via* NOD2 rescuing symptoms in a tri-nitrobenzene sulfonic acid (TNBS) -induced colitis murine model (143). Enteral administration of *L. rhamnosus* GG decreased inflammation in the developing mouse colon, attenuating pro-inflammatory MIP-2 and TNF-α concentrations in an IL-10 receptor-dependent manner (144). In Caco-2 cells *L. plantarum* WCSF1 has been shown to enhance ZO-1 trafficking to TJ regions in a toll-like receptor (TLR)-2-dependent manner (65). In a porcine intestinal cell line, *L. rhamnosus* GG ATCC 7469 pre-treatment increased ZO-1 and occludin protein expression in a TLR-2-dependent mechanism and also attenuated enterotoxigenic *E. coli* –induced increases in TNF-α *via* a partly TLR-2-mediated mechanism (145).

*Lactobacilli* may interact with enterocytes, DCs, Th1, Th2 and Treg cells in their immunomodulatory capacity in the intestine. Studies *in vitro* and *in vivo* demonstrated that *L. paracasei* and *L. acidophilus* strains induced early innate and adaptive immune responses in developing mice and rats in terms of phagocytosis, polymorphonuclear cell recruitment and TNF-α, IL-6, IL-10, IFN-γ production in a TLR-dependent mechanism (146). Homogenates prepared from several probiotics including *L. rhamnosus* GG ATCC53103, *L. rhamnosus* LC-705, *L. acidophilus* NCFB-Lb1748, and *L. bulgaricus* ATCC 11842 have demonstrated the ability to suppress peripheral blood mononuclear cell proliferation and *L. acidophilus* homogenates also down-regulated expression of IL-2 and IL-4 (147). In a mouse model of colitis where IL-10-deficient mice were infected with *H. hepaticus*, the combination of *L. paracasei* 1602 and *L. reuteri* 6798 reduced mucosal inflammatory cytokines TNF-α and IL-12 and also reduced intestinal inflammation (148). In an *in vitro* model, *L. sakei* LTH681 induced the inflammatory cytokines IL-1β, IL-8 and TNF-α in Caco-2 cells while *L. johnsonii* La1 failed to induce pro-inflammatory cytokines and instead induced production of anti-inflammatory TGF-β (149). Co-culture of ileal explants from patients with Crohn’s disease with *L. casei* DN-114001 and *L. bulgaricus* LB10 resulted in decreased TNF-α expression as well as decreased numbers of CD4+ T cells within the inflamed mucosa (150). CFCS from *L. acidophilus* ATCC 4356, *L. casei* ATCC 334, *L. lactis* ATCC 11454 and *L. reuteri* ATCC 55148 down-regulated IL-8 expression in human HT-29 cells and had differing strain-dependent efficacies in decreasing pro-inflammatory cytokines (IL-1β, IL-6, TNF-α) and in increasing anti-inflammatory IL-10 production in LPS-stimulated monocyte-derived macrophages (151).

Inflammatory bowel disease (IBD) is an example of an intestinal inflammatory disease which may be modulated by *Lactobacilli* probiotics. IBD is a chronic, relapsing and remitting disorder characterised by inflammation of the GI tract with two main classifications: Crohn’s disease and ulcerative colitis. Although the cause of IBD is unclear, dysbiosis of the GI microbiota is a feature of the disorder and it is believed probiotics may have a therapeutic benefit by restoring microbial balance and also by immunomodulation (152). Data from both *in vitro* and *in vivo* studies in animal models of colitis are extremely promising in terms of reducing inflammatory markers and decreasing colitis severity (153–155), however the same cannot be said for clinical trials of probiotics in IBD. Although it would appear that probiotics have beneficial effects in inducing remission and increasing remission times in UC (156) this has not yet been demonstrated for CD (157). A meta-analysis recently showed that *L. rhamnosus* GG displayed no beneficial effects in IBD patients, though VSL#3 (a combination of eight lactic acid bacteria strains - of which four are *Lactobacilli*) was better than placebo in terms of a higher remission rate and lower relapse rate (158). Similarly, another recent meta-analysis and systematic review concluded that a combination of *Lactobacillus* probiotics and prebiotics were effective in UC, although probiotics in general were not effective in CD (159). Further randomised, placebo controlled, clinical trials will be required to clarify the role of *Lactobacilli* in IBD and to elucidate the most beneficial strain, dose, and mode of administration.

## Conclusion and Future Perspectives

There is increasing evidence to suggest that commercial and clinical use of probiotics is outpacing proven science. A recent study in healthy human subjects given probiotic supplements indicated that the colonisation of the GI tract featured person, region and strain -specific differences. In some individuals colonisation did not occur with the GI tract demonstrating colonisation resistance to the probiotics. The authors conclude that considering the transient, individualised effect of probiotics, the development of new personalised probiotic approaches is merited (160).

Despite the ever-increasing prevalence of probiotic use, there are also many limitations and unknowns (161–163). Data from research trials on efficacy of probiotics in the treatment and prevention of disease can often have conflicting results with similar studies pointing to opposing conclusions. These confusing data are somewhat to be expected and may be accounted for given the extremely complex nature of host – probiotic – microbiota interactions. One must allow for the unique individual differences in human microbiota composition, due to age, health, diet etc., which may affect the response to the intervention and may even account for adverse effects. Risks associated with probiotic use are generally concerned with the safety of vulnerable patient cohort such as the elderly or the immunocompromised. Thorough elucidation of mechanistic properties and host interactions will required in order to determine the probiotic strains and required intake levels required to achieve the desired health outcomes. It is also of note particularly for probiotic use in healthy individuals, and indeed for mechanisms requiring microbe-host interaction, that evidence indicates that probiotics are unlikely to be capable of maintaining colonisation in the host with any differences in microbiota composition being transient and dependent on continued probiotic intake. In terms of study design, it is often the case that mechanistic observations are founded in *in vitro* cell populations which cannot give the full picture of host and microbiota interactions. These are not always supported by *in vivo* observations in animal models which themselves may be flawed given incompatibilities or inconsistencies between human and animal microbiomes. On top of this the variety of available and potential new probiotics is vast and, as we have seen, beneficial effects can be species or strain specific and may require combination with other probiotics or prebiotics to be effective. Additionally, it is often the case that probiotic trials are initiated and funded by components of the probiotic industry who have commercial interests and may have a motive to downplay adverse effects. Although systematic reviews and meta-analyses of existing studies go some way in trying to overcome biased or underpowered research and allow for observation of overall trends, they are not themselves immune from the introduction of bias. Large, long-term, multicentre randomised controlled trials of probiotics chosen based on mechanistic information with specific beneficial outcomes for specific human cohorts in mind and involving collaborations with non-affiliated groups should be the aim to truly separate the good from the ineffective or bad.

It is clear that we have a long way to go in understanding all of the complexities of the microbiota and the effects of probiotic bacteria for health. Far more in-depth clinical testing will be required in order to substantiate the health claims of commercially available probiotic health supplements. Further elucidation of the modes of action of beneficial probiotics in clearly defined subsets of populations will hopefully allow us to make better predictions about efficacy, improve clinical trial design and enable improvement in development of probiotic health strategies. Expansion in the field of bacterial-derived products i.e. postbiotics signals a more precise, effective and safer future for the probiotic health market. In the interim, those looking to improve their overall health by enhancing their GI microbial complexity might find it more advantageous to focus on consuming a healthy varied diet of grains, fruit, vegetables and fermented foods such as miso, nattō, kimchi and sauerkraut.

## Author Contributions

Writing—original draft preparation, ED; writing—review and editing, ED and SC; Conceptualization, ED and SC; Funding acquisition, SC. All authors have read and agreed to the published version of the manuscript.

## Funding

Funding for the Corr Lab is provided by Science Foundation Ireland [grant 19/FFP/6499].

## Conflict of Interest

The authors declare that the research was conducted in the absence of any commercial or financial relationships that could be construed as a potential conflict of interest.

## Publisher’s Note

All claims expressed in this article are solely those of the authors and do not necessarily represent those of their affiliated organizations, or those of the publisher, the editors and the reviewers. Any product that may be evaluated in this article, or claim that may be made by its manufacturer, is not guaranteed or endorsed by the publisher.

## References

[1] Metchnikoff E. The Prolongation of Life; Optimistic Studies. London: Heinemann (1907).

[2] Hammes WP Hertel C. Lactobacillus. In: Whitman WB Rainey F Kämpfer P Trujillo M Chun J DeVos P, editors. Bergey's Manual of Systematics of Archaea and Bacteria. (Hoboken USA: John Wiley & Sons, Inc.) In Association With Bergey’s Manual Trust (2015). p. 1–76. doi: 10.1002/9781118960608.gbm00604

[3] Ibrahim SA. Lactic Acid Bacteria: Lactobacillus Spp.: Other Species. Reference Module in Food Science: Elsevier (2016).

[4] Zheng J Wittouck S Salvetti E Franz C Harris HMB Mattarelli P. A Taxonomic Note on the Genus Lactobacillus: Description of 23 Novel Genera, Emended Description of the Genus Lactobacillus Beijerinck 1901, and Union of Lactobacillaceae and Leuconostocaceae. Int J Syst Evol Microbiol (2020) 70(4):2782–858. doi: 10.1099/ijsem.0.004107 32293557

[5] De Angelis M Gobbetti M. Lactobacillus SPP.: General Characteristics☆. In: Reference Module in Food Science. Amsterdam, Netherlands: Elsevier (2016).

[6] Heeney DD Gareau MG Marco ML. Intestinal Lactobacillus in Health and Disease, a Driver or Just Along for the Ride? Curr Opin Biotechnol (2018) 49:140–7. doi: 10.1016/j.copbio.2017.08.004 PMC5808898 28866243

[7] Matos RC Leulier F. Lactobacilli-Host Mutualism: "Learning on the Fly". Microb Cell Fact (2014) 13 Suppl 1(Suppl 1):S6–S. doi: 10.1186/1475-2859-13-S1-S6 PMC4155823 25186369

[8] FAO/WHO. Guidelines for the Evaluation of Probiotics in Food.: Food and Agriculture Organization of the United Nations and World Health Organization Working Group Report. London, Ontario, Canada: Joint FAO/WHO Working Group (2002).

[9] Hill C Guarner F Reid G Gibson GR Merenstein DJ Pot B. The International Scientific Association for Probiotics and Prebiotics Consensus Statement on the Scope and Appropriate Use of the Term Probiotic. Nat Rev Gastroenterol Hepatol (2014) 11(8):506–14. doi: 10.1038/nrgastro.2014.66 24912386

[10] Probiotics Market Size, Share & Trends Analysis Report By Product (Food & Beverages, Dietary Supplements), By Ingredient (Bacteria, Yeast), By End Use (Human, Animal), By Distribution Channel, And Segment Forecasts, 2021 - 2028: Grand View Research (2021). Available at: https://www.grandviewresearch.com/industry-analysis/probiotics-market.

[11] George Kerry R Patra JK Gouda S Park Y Shin H-S Das G. Benefaction of Probiotics for Human Health: A Review. J Food Drug Anal (2018) 26(3):927–39. doi: 10.1016/j.jfda.2018.01.002 PMC9303019 29976412

[12] Tang X Zhao J. Commercial Strains of Lactic Acid Bacteria With Health Benefits. In: Chen W, editor. Lactic Acid Bacteria: Omics and Functional Evaluation. Singapore: Springer Singapore (2019). p. 297–369.

[13] Binda S Hill C Johansen E Obis D Pot B Sanders ME. Criteria to Qualify Microorganisms as "Probiotic" in Foods and Dietary Supplements. Front Microbiol (2020) 11:1662. doi: 10.3389/fmicb.2020.01662 32793153 PMC7394020

[14] Reid G Anukam K Koyama T. Probiotic Products in Canada With Clinical Evidence: What can Gastroenterologists Recommend? Can J Gastroenterol (2008) 22(2):169–75. doi: 10.1155/2008/843892 PMC2659138 18299736

[15] Montalban-Arques A De Schryver P Bossier P Gorkiewicz G Mulero V Gatlin DM 3rd. Selective Manipulation of the Gut Microbiota Improves Immune Status in Vertebrates. Front Immunol (2015) 6:512. doi: 10.3389/fimmu.2015.00512 26500650 PMC4598590

[16] Dronkers TMG Ouwehand AC Rijkers GT. Global Analysis of Clinical Trials With Probiotics. Heliyon (2020) 6(7):e04467. doi: 10.1016/j.heliyon.2020.e04467 32715136 PMC7371762

[17] Tsai YT Cheng PC Pan TM. The Immunomodulatory Effects of Lactic Acid Bacteria for Improving Immune Functions and Benefits. Appl Microbiol Biotechnol (2012) 96(4):853–62. doi: 10.1007/s00253-012-4407-3 23001058

[18] Capurso L. Thirty Years of Lactobacillus Rhamnosus GG: A Review. . J Clin Gastroenterol (2019) 53(Suppl 1):S1–41. doi: 10.1097/MCG.0000000000001170 30741841

[19] Goldenberg JZ Yap C Lytvyn L Lo CKF Beardsley J Mertz D. Probiotics for the Prevention of Clostridium Difficile-Associated Diarrhea in Adults and Children. Cochrane Database Syst Rev (2017) 12. doi: 10.1002/14651858.CD006095.pub4 PMC6486212 29257353

[20] Sung JJY Wong SH. What Is Unknown in Using Microbiota as a Therapeutic? J Gastroenterol Hepatol (2022) 37(1):39–44. doi: 10.1111/jgh.15716 34668228

[21] Kothari D Patel S Kim S-K. Probiotic Supplements Might Not be Universally-Effective and Safe: A Review. Biomed Pharmacother (2019) 111:537–47. doi: 10.1016/j.biopha.2018.12.104 30597307

[22] Committee ES Hardy A Benford D Halldorsson T Jeger MJ Knutsen HK. Guidance on the Assessment of the Biological Relevance of Data in Scientific Assessments. EFSA J (2017) 15(8):e04970. doi: 10.2903/j.efsa.2017.4970 32625631 PMC7010076

[23] Kleerebezem M Binda S Bron PA Gross G Hill C van Hylckama Vlieg JET. Understanding Mode of Action can Drive the Translational Pipeline Towards More Reliable Health Benefits for Probiotics. Curr Opin Biotechnol (2019) 56:55–60. doi: 10.1016/j.copbio.2018.09.007 30296737

[24] Nataraj BH Ali SA Behare PV Yadav H. Postbiotics-Parabiotics: The New Horizons in Microbial Biotherapy and Functional Foods. Microb Cell Fact (2020) 19(1):168. doi: 10.1186/s12934-020-01426-w 32819443 PMC7441679

[25] Salminen S Collado MC Endo A Hill C Lebeer S Quigley EMM. The International Scientific Association of Probiotics and Prebiotics (ISAPP) Consensus Statement on the Definition and Scope of Postbiotics. Nat Rev Gastroenterol Hepatol (2021) 18(9):649–67. doi: 10.1038/s41575-021-00440-6 PMC8387231 33948025

[26] Piqué N Berlanga M Miñana-Galbis D. Health Benefits of Heat-Killed (Tyndallized) Probiotics: An Overview. Int J Mol Sci (2019) 20(10):2534. doi: 10.3390/ijms20102534 PMC6566317 31126033

[27] Teame T Wang A Xie M Zhang Z Yang Y Ding Q. Paraprobiotics and Postbiotics of Probiotic Lactobacilli, Their Positive Effects on the Host and Action Mechanisms: A Review. . Front Nutr (2020) 7:570344. doi: 10.3389/fnut.2020.570344 33195367 PMC7642493

[28] Vicariotto F Mogna L Del Piano M. Effectiveness of the Two Microorganisms Lactobacillus Fermentum LF15 and Lactobacillus Plantarum LP01, Formulated in Slow-Release Vaginal Tablets, in Women Affected by Bacterial Vaginosis: A Pilot Study. J Clin Gastroenterol (2014) 48 Suppl 1:S106–12. doi: 10.1097/MCG.0000000000000226 25291116

[29] Huang R Ning H Shen M Li J Zhang J Chen X. Probiotics for the Treatment of Atopic Dermatitis in Children: A Systematic Review and Meta-Analysis of Randomized Controlled Trials. Front Cell Infect Microbiol (2017) 7:392. doi: 10.3389/fcimb.2017.00392 28932705 PMC5592329

[30] Zhang H Yeh C Jin Z Ding L Liu BY Zhang L. Prospective Study of Probiotic Supplementation Results in Immune Stimulation and Improvement of Upper Respiratory Infection Rate. Synth Syst Biotechnol (2018) 3(2):113–20. doi: 10.1016/j.synbio.2018.03.001 PMC5995450 29900424

[31] Vancamelbeke M Vermeire S. The Intestinal Barrier: A Fundamental Role in Health and Disease. Expert Rev Gastroenterol Hepatol (2017) 11(9):821–34. doi: 10.1080/17474124.2017.1343143 PMC6104804 28650209

[32] Boirivant M Strober W. The Mechanism of Action of Probiotics. Curr Opin Gastroenterol (2007) 23(6):679–92. doi: 10.1097/MOG.0b013e3282f0cffc 17906447

[33] Plaza-Diaz J Ruiz-Ojeda FJ Gil-Campos M Gil A. Mechanisms of Action of Probiotics. Adv Nutr (Bethesda Md) (2019) 10(suppl_1):S49–s66. doi: 10.1093/advances/nmy063 PMC6363529 30721959

[34] Sartor RB. Probiotic Therapy of Intestinal Inflammation and Infections. Curr Opin Gastroenterol (2005) 21(1):44–50. 15687884

[35] Panpetch W Phuengmaung P Cheibchalard T Somboonna N Leelahavanichkul A Tumwasorn S. Lacticaseibacillus Casei Strain T21 Attenuates Clostridioides Difficile Infection in a Murine Model Through Reduction of Inflammation and Gut Dysbiosis With Decreased Toxin Lethality and Enhanced Mucin Production. Front Microbiol (2021) 12:745299. doi: 10.3389/fmicb.2021.745299 34925261 PMC8672038

[36] Shekels LL Lyftogt CT Ho SB. Bile Acid-Induced Alterations of Mucin Production in Differentiated Human Colon Cancer Cell Lines. Int J Biochem Cell Biol (1996) 28(2):193–201. doi: 10.1016/1357-2725(95)00125-5 8729006

[37] Mattar AF Teitelbaum DH Drongowski RA Yongyi F Harmon CM Coran AG. Probiotics Up-Regulate MUC-2 Mucin Gene Expression in a Caco-2 Cell-Culture Model. Pediatr Surg Int (2002) 18(7):586–90. doi: 10.1007/s00383-002-0855-7 12471471

[38] Martín R Chamignon C Mhedbi-Hajri N Chain F Derrien M Escribano-Vázquez U. The Potential Probiotic Lactobacillus Rhamnosus CNCM I-3690 Strain Protects the Intestinal Barrier by Stimulating Both Mucus Production and Cytoprotective Response. Sci Rep (2019) 9(1):5398. doi: 10.1038/s41598-019-41738-5 30931953 PMC6443702

[39] Ahl D Liu H Schreiber O Roos S Phillipson M Holm L. Lactobacillus Reuteri Increases Mucus Thickness and Ameliorates Dextran Sulphate Sodium-Induced Colitis in Mice. Acta Physiol (2016) 217(4):300–10. doi: 10.1111/apha.12695 27096537

[40] Caballero-Franco C Keller K De Simone C Chadee K. The VSL3 Probiotic Formula Induces Mucin Gene Expression and Secretion in Colonic Epithelial Cells. Am J Physiol Gastrointest Liver Physiol (2007) 292(1):G315–22. doi: 10.1152/ajpgi.00265.2006 16973917

[41] Gaudier E Michel C Segain JP Cherbut C Hoebler C. The VSL# 3 Probiotic Mixture Modifies Microflora But Does Not Heal Chronic Dextran-Sodium Sulfate-Induced Colitis or Reinforce the Mucus Barrier in Mice. J Nutr (2005) 135(12):2753–61. doi: 10.1093/jn/135.12.2753 16317116

[42] Schlee M Harder J Köten B Stange EF Wehkamp J Fellermann K. Probiotic Lactobacilli and VSL3 Induce Enterocyte Beta-Defensin 2. Clin Exp Immunol (2008) 151(3):528–35. doi: 10.1111/j.1365-2249.2007.03587.x PMC2276967 18190603

[43] Terada T Nii T Isobe N Yoshimura Y. Effects of Probiotics Lactobacillus Reuteri and Clostridium Butyricum on the Expression of Toll-Like Receptors, Pro- and Anti-Inflammatory Cytokines, and Antimicrobial Peptides in Broiler Chick Intestine. J Poult Sci (2020) 57(4):310–8. doi: 10.2141/jpsa.0190098 PMC7596031 33132732

[44] Alakomi HL Skytta E Saarela M Mattila-Sandholm T Latva-Kala K Helander IM. Lactic Acid Permeabilizes Gram-Negative Bacteria by Disrupting the Outer Membrane. Appl Environ Microbiol (2000) 66(5):2001–5. doi: 10.1128/AEM.66.5.2001-2005.2000 PMC101446 10788373

[45] Atassi F Servin AL. Individual and Co-Operative Roles of Lactic Acid and Hydrogen Peroxide in the Killing Activity of Enteric Strain Lactobacillus Johnsonii NCC933 and Vaginal Strain Lactobacillus Gasseri KS120.1 against enteric, uropathogenic and vaginosis-associated pathogens. FEMS Microbiol Lett (2010) 304(1):29–38. doi: 10.1111/j.1574-6968.2009.01887.x 20082639

[46] Dicks LMT Dreyer L Smith C Van Staden Ad. A Review: The Fate of Bacteriocins in the Human Gastro-Intestinal Tract: Do They Cross the Gut–Blood Barrier? Front Microbiol (2018) 9(2297). doi: 10.3389/fmicb.2018.02297 PMC6173059 30323796

[47] Jack RW Tagg JR Ray B. Bacteriocins of Gram-Positive Bacteria. Microbiol Rev (1995) 59(2):171–200. doi: 10.1128/mr.59.2.171-200.1995 7603408 PMC239359

[48] Feng Y Wang Y Wang P Huang Y Wang F. Short-Chain Fatty Acids Manifest Stimulative and Protective Effects on Intestinal Barrier Function Through the Inhibition of NLRP3 Inflammasome and Autophagy. Cell Physiol Biochem (2018) 49(1):190–205. doi: 10.1159/000492853 30138914

[49] Todorov SD. Bacteriocins From Lactobacillus Plantarum - Production, Genetic Organization and Mode of Action: Produção, Organização Genética E Modo De Ação. Braz J Microbiol (2009) 40(2):209–21. doi: 10.1590/S1517-83822009000200001 PMC3769724 24031346

[50] Corr SC Li Y Riedel CU O'Toole PW Hill C Gahan CG. Bacteriocin Production as a Mechanism for the Antiinfective Activity of Lactobacillus Salivarius UCC118. Proc Natl Acad Sci USA (2007) 104(18):7617–21. doi: 10.1073/pnas.0700440104 PMC1863472 17456596

[51] O' Shea E O' Connor P Raftis EJ O' Toole P Stanton C Cotter PD. Subspecies Diversity in Bacteriocin Production by Intestinal Lactobacillus Salivarius Strains. Gut Microbes (2012) 3(5):468–73. doi: 10.4161/gmic.21417 PMC3466502 22892690

[52] Cerutti A Rescigno M. The Biology of Intestinal Immunoglobulin A Responses. Immunity (2008) 28(6):740–50. doi: 10.1016/j.immuni.2008.05.001 PMC3057455 18549797

[53] Pabst O Slack E. IgA and the Intestinal Microbiota: The Importance of Being Specific. Mucosal Immunol (2020) 13(1):12–21. doi: 10.1038/s41385-019-0227-4 31740744 PMC6914667

[54] Peterson DA McNulty NP Guruge JL Gordon JI. IgA Response to Symbiotic Bacteria as a Mediator of Gut Homeostasis. Cell Host Microbe (2007) 2(5):328–39. doi: 10.1016/j.chom.2007.09.013 18005754

[55] Rhee KJ Sethupathi P Driks A Lanning DK Knight KL. Role of Commensal Bacteria in Development of Gut-Associated Lymphoid Tissues and Preimmune Antibody Repertoire. J Immunol (2004) 172(2):1118–24. doi: 10.4049/jimmunol.172.2.1118 14707086

[56] Pasquier B Launay P Kanamaru Y Moura IC Pfirsch S Ruffie C. Identification of FcalphaRI as an Inhibitory Receptor That Controls Inflammation: Dual Role of FcRgamma ITAM. Immunity (2005) 22(1):31–42. doi: 10.1016/S1074-7613(04)00377-2 15664157

[57] Yanagibashi T Hosono A Oyama A Tsuda M Suzuki A Hachimura S. IgA Production in the Large Intestine is Modulated by a Different Mechanism Than in the Small Intestine: Bacteroides Acidifaciens Promotes IgA Production in the Large Intestine by Inducing Germinal Center Formation and Increasing the Number of IgA+ B Cells. Immunobiology (2013) 218(4):645–51. doi: 10.1016/j.imbio.2012.07.033 22940255

[58] Arai S Iwabuchi N Takahashi S J-z X Abe F Hachimura S. Orally Administered Heat-Killed Lactobacillus Paracasei MCC1849 Enhances Antigen-Specific IgA Secretion and Induces Follicular Helper T Cells in Mice. PloS One (2018) 13(6):e0199018. doi: 10.1371/journal.pone.0199018 29897995 PMC5999281

[59] Sakai F Hosoya T Ono-Ohmachi A Ukibe K Ogawa A Moriya T. Lactobacillus Gasseri SBT2055 Induces TGF-β Expression in Dendritic Cells and Activates TLR2 Signal to Produce IgA in the Small Intestine. PloS One (2014) 9(8):e105370. doi: 10.1371/journal.pone.0105370 25144744 PMC4140756

[60] Kikuchi Y Kunitoh-Asari A Hayakawa K Imai S Kasuya K Abe K. Oral Administration of Lactobacillus Plantarum Strain AYA Enhances IgA Secretion and Provides Survival Protection Against Influenza Virus Infection in Mice. PloS One (2014) 9(1):e86416. doi: 10.1371/journal.pone.0086416 24466081 PMC3899257

[61] Kusumo PD Bela B Wibowo H Munasir Z Surono IS. Lactobacillus Plantarum IS-10506 Supplementation Increases Faecal Siga and Immune Response in Children Younger Than Two Years. Benefic Microbes (2019) 10(3):245–52. doi: 10.3920/BM2017.0178 30694099

[62] Ulluwishewa D Anderson RC McNabb WC Moughan PJ Wells JM Roy NC. Regulation of Tight Junction Permeability by Intestinal Bacteria and Dietary Components. J Nutr (2011) 141(5):769–76. doi: 10.3945/jn.110.135657 21430248

[63] Orlando A Linsalata M Notarnicola M Tutino V Russo F. Lactobacillus GG Restoration of the Gliadin Induced Epithelial Barrier Disruption: The Role of Cellular Polyamines. BMC Microbiol (2014) 14:19. doi: 10.1186/1471-2180-14-19 24483336 PMC3911798

[64] Johnson-Henry KC Donato KA Shen-Tu G Gordanpour M Sherman PM. Lactobacillus Rhamnosus Strain GG Prevents Enterohemorrhagic Escherichia Coli O157:H7-Induced Changes in Epithelial Barrier Function. Infect Immunity (2008) 76(4):1340–8. doi: 10.1128/IAI.00778-07 PMC2292865 18227169

[65] Karczewski J Troost FJ Konings I Dekker J Kleerebezem M Brummer RJ. Regulation of Human Epithelial Tight Junction Proteins by Lactobacillus Plantarum In Vivo and Protective Effects on the Epithelial Barrier. Am J Physiol Gastrointest Liver Physiol (2010) 298(6):G851–9. doi: 10.1152/ajpgi.00327.2009 20224007

[66] Qin H Zhang Z Hang X Jiang YL. Plantarum Prevents Enteroinvasive Escherichia Coli-Induced Tight Junction Proteins Changes in Intestinal Epithelial Cells. BMC Microbiol (2009) 9:63. doi: 10.1186/1471-2180-9-63 19331693 PMC2674056

[67] Anderson RC Cookson AL McNabb WC Park Z McCann MJ Kelly WJ. Lactobacillus Plantarum MB452 Enhances the Function of the Intestinal Barrier by Increasing the Expression Levels of Genes Involved in Tight Junction Formation. BMC Microbiol (2010) 10(1):316. doi: 10.1186/1471-2180-10-316 21143932 PMC3004893

[68] Cario E Gerken G Podolsky DK. Toll-Like Receptor 2 Enhances ZO-1-Associated Intestinal Epithelial Barrier Integrity *via* Protein Kinase C. Gastroenterology (2004) 127(1):224–38. doi: 10.1053/j.gastro.2004.04.015 15236188

[69] Hummel S Veltman K Cichon C Sonnenborn U Schmidt MA. Differential Targeting of the E-Cadherin/beta-Catenin Complex by Gram-Positive Probiotic Lactobacilli Improves Epithelial Barrier Function. Appl Environ Microbiol (2012) 78(4):1140–7. doi: 10.1128/AEM.06983-11 PMC3272997 22179242

[70] Mujagic Z de Vos P Boekschoten MV Govers C Pieters H-JHM de Wit NJW. The Effects of Lactobacillus Plantarum on Small Intestinal Barrier Function and Mucosal Gene Transcription; a Randomized Double-Blind Placebo Controlled Trial. Sci Rep (2017) 7:40128. doi: 10.1038/srep40128 28045137 PMC5206730

[71] Celebioglu HU Svensson B. Dietary Nutrients, Proteomes, and Adhesion of Probiotic Lactobacilli to Mucin and Host Epithelial Cells. Microorganisms (2018) 6(3):90. doi: 10.3390/microorganisms6030090 PMC6163540 30134518

[72] Yadav AK Tyagi A Kumar A Panwar S Grover S Saklani AC. Adhesion of Lactobacilli and Their Anti-Infectivity Potential. Crit Rev Food Sci Nutr (2017) 57(10):2042–56. doi: 10.1080/10408398.2014.918533 25879917

[73] Jensen H Roos S Jonsson H Rud I Grimmer S van Pijkeren JP. Role of Lactobacillus Reuteri Cell and Mucus-Binding Protein A (CmbA) in Adhesion to Intestinal Epithelial Cells and Mucus In Vitro. Microbiol (Read) (2014) 160(Pt 4):671–81. doi: 10.1099/mic.0.073551-0 PMC7336543 24473252

[74] Hynönen U Palva A. Lactobacillus Surface Layer Proteins: Structure, Function and Applications. Appl Microbiol Biotechnol (2013) 97(12):5225–43. doi: 10.1007/s00253-013-4962-2 PMC3666127 23677442

[75] Prado Acosta M Geoghegan EM Lepenies B Ruzal S Kielian M Martinez MG. Surface (S) Layer Proteins of Lactobacillus Acidophilus Block Virus Infection *via* DC-SIGN Interaction. Front Microbiol (2019) 10. doi: 10.3389/fmicb.2019.00810 PMC6477042 31040840

[76] Tytgat HLP Douillard FP Reunanen J Rasinkangas P Hendrickx APA Laine PK. Lactobacillus Rhamnosus GG Outcompetes Enterococcus Faecium *via* Mucus-Binding Pili: Evidence for a Novel and Heterospecific Probiotic Mechanism. Appl Environ Microbiol (2016) 82(19):5756–62. doi: 10.1128/AEM.01243-16 PMC5038030 27422834

[77] Jeffery CJ. Protein Moonlighting: What Is It, and Why Is It Important? Philosophical Transactions of the Royal Society of London Series B. Biol Sci (2018) 373(1738):20160523. doi: 10.1098/rstb.2016.0523 PMC5717523 29203708

[78] Saad N Urdaci M Vignoles C Chaignepain S Tallon R Schmitter JM. Lactobacillus Plantarum 299v Surface-Bound GAPDH: A New Insight Into Enzyme Cell Walls Location. J Microbiol Biotechnol (2009) 19(12):1635–43. doi: 10.4014/jmb.0902.0102 20075631

[79] Patel DK Shah KR Pappachan A Gupta S Singh DD. Cloning, Expression and Characterization of a Mucin-Binding GAPDH From Lactobacillus Acidophilus. Int J Biol Macromol (2016) 91:338–46. doi: 10.1016/j.ijbiomac.2016.04.041 27180300

[80] Zhang W-M Wang H-F Gao K Wang C Liu L Liu J-X. Lactobacillus Reuteri Glyceraldehyde-3-Phosphate Dehydrogenase Functions in Adhesion to Intestinal Epithelial Cells. Can J Microbiol (2015) 61(5):373–80. doi: 10.1139/cjm-2014-0734 25867279

[81] Ramiah K van Reenen CA Dicks LM. Surface-Bound Proteins of Lactobacillus Plantarum 423 That Contribute to Adhesion of Caco-2 Cells and Their Role in Competitive Exclusion and Displacement of Clostridium Sporogenes and Enterococcus Faecalis. Res Microbiol (2008) 159(6):470–5. doi: 10.1016/j.resmic.2008.06.002 18619532

[82] Sherman PM Johnson-Henry KC Yeung HP Ngo PS Goulet J Tompkins TA. Probiotics Reduce Enterohemorrhagic Escherichia Coli O157:H7- and Enteropathogenic E. coli O127:H6-induced changes in polarized T84 epithelial cell monolayers by reducing bacterial adhesion and cytoskeletal rearrangements. Infect Immun (2005) 73(8):5183–8. doi: 10.1128/IAI.73.8.5183-5188.2005 PMC1201237 16041036

[83] Singh TP Kaur G Kapila S Malik RK. Antagonistic Activity of Lactobacillus Reuteri Strains on the Adhesion Characteristics of Selected Pathogens. Front Microbiol (2017) 8(486). doi: 10.3389/fmicb.2017.00486 PMC5359300 28377765

[84] Hirano J Yoshida T Sugiyama T Koide N Mori I Yokochi T. The Effect of Lactobacillus Rhamnosus on Enterohemorrhagic Escherichia Coli Infection of Human Intestinal Cells In Vitro. Microb Immunol (2003) 47(6):405–9. doi: 10.1111/j.1348-0421.2003.tb03377.x 12906100

[85] Zareie M Johnson-Henry K Jury J Yang PC Ngan BY McKay DM. Probiotics Prevent Bacterial Translocation and Improve Intestinal Barrier Function in Rats Following Chronic Psychological Stress. Gut (2006) 55(11):1553–60. doi: 10.1136/gut.2005.080739 PMC1860130 16638791

[86] Luyer MD Buurman WA Hadfoune M Speelmans G Knol J Jacobs JA. Strain-Specific Effects of Probiotics on Gut Barrier Integrity Following Hemorrhagic Shock. Infect Immun (2005) 73(6):3686–92. doi: 10.1128/IAI.73.6.3686-3692.2005 PMC1111872 15908398

[87] Schoster A Kokotovic B Permin A Pedersen PD Dal Bello F Guardabassi L. *In Vitro* Inhibition of Clostridium Difficile and Clostridium Perfringens by Commercial Probiotic Strains. Anaerobe (2013) 20:36–41. doi: 10.1016/j.anaerobe.2013.02.006 23471038

[88] Saint-Cyr MJ Haddad N Taminiau B Poezevara T Quesne S Amelot M. Use of the Potential Probiotic Strain Lactobacillus Salivarius SMXD51 to Control Campylobacter Jejuni in Broilers. Int J Food Microbiol (2017) 247:9–17. doi: 10.1016/j.ijfoodmicro.2016.07.003 27432696

[89] Carter A Adams M La Ragione RM Woodward MJ. Colonisation of Poultry by Salmonella Enteritidis S1400 is Reduced by Combined Administration of Lactobacillus Salivarius 59 and Enterococcus Faecium PXN-33. Vet Microbiol (2017) 199:100–7. doi: 10.1016/j.vetmic.2016.12.029 28110775

[90] Chingwaru W Vidmar J. Potential of Zimbabwean Commercial Probiotic Products and Strains of Lactobacillus Plantarum as Prophylaxis and Therapy Against Diarrhoea Caused by Escherichia Coli in Children. Asian Pac J Trop Med (2017) 10(1):57–63. doi: 10.1016/j.apjtm.2016.12.009 28107866

[91] Sikorska H Smoragiewicz W. Role of Probiotics in the Prevention and Treatment of Meticillin-Resistant Staphylococcus Aureus Infections. Int J Antimicrob Agents (2013) 42(6):475–81. doi: 10.1016/j.ijantimicag.2013.08.003 24071026

[92] De Montijo-Prieto S Moreno E Bergillos-Meca T Lasserrot A Ruiz-Lopez MD Ruiz-Bravo A. A Lactobacillus Plantarum Strain Isolated From Kefir Protects Against Intestinal Infection With Yersinia Enterocolitica O9 and Modulates Immunity in Mice. Res Microbiol (2015) 166(8):626–32. doi: 10.1016/j.resmic.2015.07.010 26272025

[93] Hooi JKY Lai WY Ng WK Suen MMY Underwood FE Tanyingoh D. Global Prevalence of Helicobacter Pylori Infection: Systematic Review and Meta-Analysis. Gastroenterology (2017) 153(2):420–9. doi: 10.1053/j.gastro.2017.04.022 28456631

[94] Waldum HL Kleveland PM Sørdal ØF. Helicobacter Pylori and Gastric Acid: An Intimate and Reciprocal Relationship. Ther Adv Gastroenterol (2016) 9(6):836–44. doi: 10.1177/1756283X16663395 PMC5076771 27803738

[95] Sykora J Valeckova K Amlerova J Siala K Dedek P Watkins S. Effects of a Specially Designed Fermented Milk Product Containing Probiotic Lactobacillus Casei DN-114 001 and the Eradication of H. pylori in Children: A Prospective Randomized Double-Blind Study. J Clin Gastroenterol (2005) 39(8):692–8. doi: 10.1097/01.mcg.0000173855.77191.44 16082279

[96] Sahagun-Flores JE Lopez-Pena LS De La Cruz-Ramirez Jaimes J Garcia-Bravo MS Peregrina-Gomez R De Alba-Garcia Je. [Eradication of Helicobacter Pylori: Triple Treatment Scheme Plus Lactobacillus vs. Triple Treatment Alone]. Cirugia Y Cirujanos (2007) 75(5):333–6. 18158878

[97] Canducci F Armuzzi A Cremonini F Cammarota G Bartolozzi F Pola P. A Lyophilized and Inactivated Culture of Lactobacillus Acidophilus Increases Helicobacter Pylori Eradication Rates. Aliment Pharmacol Ther (2000) 14(12):1625–9. doi: 10.1046/j.1365-2036.2000.00885.x 11121911

[98] Cremonini F Di Caro S Covino M Armuzzi A Gabrielli M Santarelli L. Effect of Different Probiotic Preparations on Anti-Helicobacter Pylori Therapy-Related Side Effects: A Parallel Group, Triple Blind, Placebo-Controlled Study. Am J Gastroenterol (2002) 97(11):2744–9. doi: 10.1111/j.1572-0241.2002.07063.x 12425542

[99] Myllyluoma E Veijola L Ahlroos T Tynkkynen S Kankuri E Vapaatalo H. Probiotic Supplementation Improves Tolerance to Helicobacter Pylori Eradication Therapy–a Placebo-Controlled, Double-Blind Randomized Pilot Study. Aliment Pharmacol Ther (2005) 21(10):1263–72. doi: 10.1111/j.1365-2036.2005.02448.x 15882248

[100] Szajewska H Albrecht P Topczewska-Cabanek A. Randomized, Double-Blind, Placebo-Controlled Trial: Effect of Lactobacillus GG Supplementation on Helicobacter Pylori Eradication Rates and Side Effects During Treatment in Children. J Pediatr Gastroenterol Nutr (2009) 48(4):431–6. doi: 10.1097/MPG.0b013e318182e716 19330931

[101] Francavilla R Lionetti E Castellaneta SP Magista AM Maurogiovanni G Bucci N. Inhibition of Helicobacter Pylori Infection in Humans by Lactobacillus Reuteri ATCC 55730 and Effect on Eradication Therapy: A Pilot Study. Helicobacter (2008) 13(2):127–34. doi: 10.1111/j.1523-5378.2008.00593.x 18321302

[102] Pantoflickova D Corthesy-Theulaz I Dorta G Stolte M Isler P Rochat F. Favourable Effect of Regular Intake of Fermented Milk Containing Lactobacillus Johnsonii on Helicobacter Pylori Associated Gastritis. Aliment Pharmacol Ther (2003) 18(8):805–13. doi: 10.1046/j.1365-2036.2003.01675.x 14535874

[103] Gotteland M Cruchet S. Suppressive Effect of Frequent Ingestion of Lactobacillus Johnsonii La1 on Helicobacter Pylori Colonization in Asymptomatic Volunteers. J Antimicrob Chemother (2003) 51(5):1317–9. doi: 10.1093/jac/dkg227 12697639

[104] Le Moal VL Fayol-Messaoudi D Servin AL. Compound(s) Secreted by Lactobacillus Casei Strain Shirota YIT9029 Irreversibly and Reversibly Impair the Swimming Motility of Helicobacter Pylori and Salmonella Enterica Serovar Typhimurium, Respectively. Microbiol (Reading England) (2013) 159(Pt 9):1956–71. doi: 10.1099/mic.0.067678-0 23873784

[105] Coconnier MH Lievin V Hemery E Servin AL. Antagonistic Activity Against Helicobacter Infection *In Vitro* and *In Vivo* by the Human Lactobacillus Acidophilus Strain LB. Appl Environ Microbiol (1998) 64(11):4573–80. doi: 10.1128/AEM.64.11.4573-4580.1998 PMC106686 9797324

[106] Sgouras DN Panayotopoulou EG Martinez-Gonzalez B Petraki K Michopoulos S Mentis A. Lactobacillus Johnsonii La1 Attenuates Helicobacter Pylori-Associated Gastritis and Reduces Levels of Proinflammatory Chemokines in C57BL/6 Mice. Clin Diagn Lab Immunol (2005) 12(12):1378–86. doi: 10.1128/CDLI.12.12.1378-1386.2005 PMC1317072 16339060

[107] Michetti P Dorta G Wiesel PH Brassart D Verdu E Herranz M. Effect of Whey-Based Culture Supernatant of Lactobacillus Acidophilus (Johnsonii) La1 on Helicobacter Pylori Infection in Humans. Digestion (1999) 60(3):203–9. doi: 10.1159/000007660 10343133

[108] Andersen LP Rasmussen L. Helicobacter Pylori-Coccoid Forms and Biofilm Formation. FEMS Immunol Med Microbiol (2009) 56(2):112–5. doi: 10.1111/j.1574-695X.2009.00556.x 19453756

[109] Reshetnyak VI Reshetnyak TM. Significance of Dormant Forms of Helicobacter Pylori in Ulcerogenesis. World J Gastroenterol (2017) 23(27):4867–78. doi: 10.3748/wjg.v23.i27.4867 PMC5526757 28785141

[110] Avonts L De Vuyst L. Antimicrobial Potential of Probiotic Lactic Acid Bacteria. Mededelingen (Rijksuniversiteit Te Gent Fakulteit Van Landbouwkundige En Toegepaste Biologische Wetenschappen) (2001) 66(3b):543–50. 15954651

[111] Ottemann KM Lowenthal AC. Helicobacter Pylori Uses Motility for Initial Colonization and to Attain Robust Infection. Infect Immunity (2002) 70(4):1984–90. doi: 10.1128/IAI.70.4.1984-1990.2002 PMC127824 11895962

[112] Isobe H Nishiyama A Takano T Higuchi W Nakagawa S Taneike I. Reduction of Overall Helicobacter Pylori Colonization Levels in the Stomach of Mongolian Gerbil by Lactobacillus Johnsonii La1 (LC1) and its *In Vitro* Activities Against H. pylori Motility and Adherence. Biosci Biotechnol Biochem (2012) 76(4):850–2. doi: 10.1271/bbb.110921 22484956

[113] Barbut F Meynard JL. Managing Antibiotic Associated Diarrhoea. BMJ (Clinical Res ed) (2002) 324(7350):1345–6. doi: 10.1136/bmj.324.7350.1345 PMC1123310 12052785

[114] Zhou FF Wu S Klena JD Huang HH. Clinical Characteristics of Clostridium Difficile Infection in Hospitalized Patients With Antibiotic-Associated Diarrhea in a University Hospital in China. Eur J Clin Microbiol Infect Dis (2014) 33(10):1773–9. doi: 10.1007/s10096-014-2132-9 PMC4674785 24820293

[115] Mekonnen SA Merenstein D Fraser CM Marco ML. Molecular Mechanisms of Probiotic Prevention of Antibiotic-Associated Diarrhea. Curr Opin Biotechnol (2020) 61:226–34. doi: 10.1016/j.copbio.2020.01.005 PMC7096272 32087535

[116] Moayyedi P Yuan Y Baharith H Ford AC. Faecal Microbiota Transplantation for Clostridium Difficile-Associated Diarrhoea: A Systematic Review of Randomised Controlled Trials. Med J Aust (2017) 207(4):166–72. doi: 10.5694/mja17.00295 28814204

[117] Kale-Pradhan PB Jassal HK Wilhelm SM. Role of Lactobacillus in the Prevention of Antibiotic-Associated Diarrhea: A Meta-Analysis. Pharmacotherapy (2010) 30(2):119–26. doi: 10.1592/phco.30.2.119 20099986

[118] Cai J Zhao C Du Y Zhang Y Zhao M Zhao Q. Comparative Efficacy and Tolerability of Probiotics for Antibiotic-Associated Diarrhea: Systematic Review With Network Meta-Analysis. United Eur Gastroenterol J (2018) 6(2):169–80. doi: 10.1177/2050640617736987 PMC5833232 29511547

[119] Szajewska H Hojsak I. Health Benefits of Lactobacillus Rhamnosus GG and Bifidobacterium Animalis Subspecies Lactis BB-12 in Children. Postgrad Med (2020) 132(5):441–51. doi: 10.1080/00325481.2020.1731214 32059116

[120] Lebeer S Vanderleyden J De Keersmaecker SC. Host Interactions of Probiotic Bacterial Surface Molecules: Comparison With Commensals and Pathogens. Nat Rev Microbiol (2010) 8(3):171–84. doi: 10.1038/nrmicro2297 20157338

[121] Wells JM. Immunomodulatory Mechanisms of Lactobacilli. Microb Cell Fact (2011) 10(1):S17. doi: 10.1186/1475-2859-10-S1-S17 21995674 PMC3231924

[122] Azad MAK Sarker M Wan D. Immunomodulatory Effects of Probiotics on Cytokine Profiles. BioMed Res Int (2018) 2018:8063647. doi: 10.1155/2018/8063647 30426014 PMC6218795

[123] Shida K Kiyoshima-Shibata J Kaji R Nagaoka M Nanno M. Peptidoglycan From Lactobacilli Inhibits Interleukin-12 Production by Macrophages Induced by Lactobacillus casei Through Toll-Like Receptor 2-Dependent and Independent Mechanisms. Immunology (2009) 128(1pt2):e858–e69. doi: 10.1111/j.1365-2567.2009.03095.x PMC2753894 19740347

[124] Kolling Y Salva S Villena J Alvarez S. Are the Immunomodulatory Properties of Lactobacillus Rhamnosus CRL1505 Peptidoglycan Common for All Lactobacilli During Respiratory Infection in Malnourished Mice? PloS One (2018) 13(3):e0194034. doi: 10.1371/journal.pone.0194034 29518131 PMC5843338

[125] Noh SY Kang SS Yun CH Han SH. Lipoteichoic Acid From Lactobacillus Plantarum Inhibits Pam2CSK4-Induced IL-8 Production in Human Intestinal Epithelial Cells. Mol Immunol (2015) 64(1):183–9. doi: 10.1016/j.molimm.2014.11.014 25481370

[126] Kim KW Kang S-S Woo S-J Park O-J Ahn KB Song K-D. Lipoteichoic Acid of Probiotic Lactobacillus Plantarum Attenuates Poly I:C-Induced IL-8 Production in Porcine Intestinal Epithelial Cells. . Front Microbiol (2017) 8:1827. doi: 10.3389/fmicb.2017.01827 28983294 PMC5613100

[127] Lebeer S Claes I Tytgat HLP Verhoeven TLA Marien E Ossowski Iv. Functional Analysis of Lactobacillus Rhamnosus GG Pili in Relation to Adhesion and Immunomodulatory Interactions With Intestinal Epithelial Cells. Appl Environ Microbiol (2012) 78(1):185–93. doi: 10.1128/AEM.06192-11 PMC3255643 22020518

[128] Bleau C Monges A Rashidan K Laverdure J-P Lacroix M Van Calsteren M-R. Intermediate Chains of Exopolysaccharides From Lactobacillus Rhamnosus RW-9595M Increase IL-10 Production by Macrophages. J Appl Microbiol (2010) 108(2):666–75. doi: 10.1111/j.1365-2672.2009.04450.x 19702865

[129] Murofushi Y Villena J Morie K Kanmani P Tohno M Shimazu T. The Toll-Like Receptor Family Protein RP105/MD1 Complex is Involved in the Immunoregulatory Effect of Exopolysaccharides From Lactobacillus Plantarum N14. Mol Immunol (2015) 64(1):63–75. doi: 10.1016/j.molimm.2014.10.027 25466614

[130] Makino S Sato A Goto A Nakamura M Ogawa M Chiba Y. Enhanced Natural Killer Cell Activation by Exopolysaccharides Derived From Yogurt Fermented With Lactobacillus Delbrueckii Ssp. Bulgaricus OLL1073R-1. J Dairy Sci (2016) 99(2):915–23. doi: 10.3168/jds.2015-10376 26686726

[131] Sadler R Cramer JV Heindl S Kostidis S Betz D Zuurbier KR. Short-Chain Fatty Acids Improve Poststroke Recovery *via* Immunological Mechanisms. J Neurosci (2020) 40(5):1162–73. doi: 10.1523/JNEUROSCI.1359-19.2019 PMC6989004 31889008

[132] Vinolo MAR Rodrigues HG Hatanaka E Sato FT Sampaio SC Curi R. Suppressive Effect of Short-Chain Fatty Acids on Production of Proinflammatory Mediators by Neutrophils. J Nutr Biochem (2011) 22(9):849–55. doi: 10.1016/j.jnutbio.2010.07.009 21167700

[133] Park JS Lee EJ Lee JC Kim WK Kim HS. Anti-Inflammatory Effects of Short Chain Fatty Acids in IFN-Gamma-Stimulated RAW 264.7 murine macrophage cells: involvement of NF-kappaB and ERK signaling pathways. Int Immunopharmacol (2007) 7(1):70–7. doi: 10.1016/j.intimp.2006.08.015 17161819

[134] Tominaga K Kamimura K Takahashi K Yokoyama J Yamagiwa S Terai S. Diversion Colitis and Pouchitis: A Mini-Review. World J Gastroenterol (2018) 24(16):1734–47. doi: 10.3748/wjg.v24.i16.1734 PMC5922993 29713128

[135] Şengül N Işık S Aslım B Uçar G Demirbağ AE. The Effect of Exopolysaccharide-Producing Probiotic Strains on Gut Oxidative Damage in Experimental Colitis. Digest Dis Sci (2011) 56(3):707–14. doi: 10.1007/s10620-010-1362-7 20683661

[136] Suo H Zhao X Qian Y Sun P Zhu K Li J. Lactobacillus Fermentum Suo Attenuates HCl/Ethanol Induced Gastric Injury in Mice Through Its Antioxidant Effects. Nutrients (2016) 8(3):155. doi: 10.3390/nu8030155 26978395 PMC4808883

[137] Uskova MA Kravchenko LV. [Antioxidant Properties of Lactic Acid Bacteria–Probiotic and Yogurt Strains]. Voprosy Pitaniia (2009) 78(2):18–23. 19514338

[138] Kleniewska P Pawliczak R. Influence of Synbiotics on Selected Oxidative Stress Parameters. Oxid Med Cell Longev (2017) 2017:9315375. doi: 10.1155/2017/9315375 28286605 PMC5327756

[139] Verma A Shukla G. Synbiotic (Lactobacillus Rhamnosus+Lactobacillus Acidophilus+Inulin) Attenuates Oxidative Stress and Colonic Damage in 1,2 Dimethylhydrazine Dihydrochloride-Induced Colon Carcinogenesis in Sprague-Dawley Rats: A Long-Term Study. Eur J Cancer Prev (2014) 23(6):550–9. doi: 10.1097/CEJ.0000000000000054 25025584

[140] Yan F Cao H Cover TL Whitehead R Washington MK Polk DB. Soluble Proteins Produced by Probiotic Bacteria Regulate Intestinal Epithelial Cell Survival and Growth. Gastroenterology (2007) 132(2):562–75. doi: 10.1053/j.gastro.2006.11.022 PMC3036990 17258729

[141] Yoda K Miyazawa K Hosoda M Hiramatsu M Yan F He F. Lactobacillus GG-Fermented Milk Prevents DSS-Induced Colitis and Regulates Intestinal Epithelial Homeostasis Through Activation of Epidermal Growth Factor Receptor. Eur J Nutr (2014) 53(1):105–15. doi: 10.1007/s00394-013-0506-x PMC4064457 23468308

[142] Tao Y Drabik KA Waypa TS Musch MW Alverdy JC Schneewind O. Soluble Factors From Lactobacillus GG Activate MAPKs and Induce Cytoprotective Heat Shock Proteins in Intestinal Epithelial Cells. Am J Physiol-Cell Physiol (2006) 290(4):C1018–C30. doi: 10.1152/ajpcell.00131.2005 16306130

[143] Macho Fernandez E Valenti V Rockel C Hermann C Pot B Boneca IG. Anti-Inflammatory Capacity of Selected Lactobacilli in Experimental Colitis is Driven by NOD2-Mediated Recognition of a Specific Peptidoglycan-Derived Muropeptide. Gut (2011) 60(8):1050–9. doi: 10.1136/gut.2010.232918 21471573

[144] Mirpuri J Sotnikov I Myers L Denning TL Yarovinsky F Parkos CA. Lactobacillus Rhamnosus (LGG) Regulates IL-10 Signaling in the Developing Murine Colon Through Upregulation of the IL-10R2 Receptor Subunit. PloS One (2012) 7(12):e51955. doi: 10.1371/journal.pone.0051955 23272193 PMC3525658

[145] Zhang W Zhu Y-H Yang J-C Yang G-Y Zhou D Wang J-F. A Selected Lactobacillus Rhamnosus Strain Promotes EGFR-Independent Akt Activation in an Enterotoxigenic Escherichia Coli K88-Infected IPEC-J2 Cell Model. PloS One (2015) 10(4):e0125717–e. doi: 10.1371/journal.pone.0125717 PMC4411159 25915861

[146] Kourelis A Zinonos I Kakagianni M Christidou A Christoglou N Yiannaki E. Validation of the Dorsal Air Pouch Model to Predict and Examine Immunostimulatory Responses in the Gut. J Appl Microbiol (2010) 108(1):274–84. doi: 10.1111/j.1365-2672.2009.04421.x 20002910

[147] Kankaanpää P Sütas Y Salminen S Isolauri E. Homogenates Derived From Probiotic Bacteria Provide Down-Regulatory Signals for Peripheral Blood Mononuclear Cells. Food Chem (2003) 83(2):269–77. doi: 10.1016/S0308-8146(03)00090-6

[148] Peña JA Rogers AB Ge Z Ng V Li SY Fox JG. Probiotic Lactobacillus Spp. Diminish Helicobacter Hepaticus-Induced Inflammatory Bowel Disease in Interleukin-10-Deficient Mice. Infect Immun (2005) 73(2):912–20. doi: 10.1128/IAI.73.2.912-920.2005 PMC547020 15664933

[149] Haller D Bode C Hammes WP Pfeifer AM Schiffrin EJ Blum S. Non-Pathogenic Bacteria Elicit a Differential Cytokine Response by Intestinal Epithelial Cell/Leucocyte Co-Cultures. Gut (2000) 47(1):79–87. doi: 10.1136/gut.47.1.79 10861268 PMC1727962

[150] Borruel N Carol M Casellas F Antolín M de Lara F Espín E. Increased Mucosal Tumour Necrosis Factor Alpha Production in Crohn's Disease can be Downregulated Ex Vivo by Probiotic Bacteria. Gut (2002) 51(5):659–64. doi: 10.1136/gut.51.5.659 PMC1773447 12377803

[151] De Marco S Sichetti M Muradyan D Piccioni M Traina G Pagiotti R. Probiotic Cell-Free Supernatants Exhibited Anti-Inflammatory and Antioxidant Activity on Human Gut Epithelial Cells and Macrophages Stimulated With LPS. Evid Based Complement Alternat Med (2018) 2018:1756308–. doi: 10.1155/2018/1756308 PMC6057331 30069221

[152] Islam SU. Clinical Uses of Probiotics. Medicine (2016) 95(5):e2658. doi: 10.1097/MD.0000000000002658 26844491 PMC4748908

[153] Alard J Peucelle V Boutillier D Breton J Kuylle S Pot B. New Probiotic Strains for Inflammatory Bowel Disease Management Identified by Combining *In Vitro* and *In Vivo* Approaches. Benefic Microbes (2018) 9(2):317–31. doi: 10.3920/BM2017.0097 29488412

[154] Le B Yang SH. Efficacy of Lactobacillus Plantarum in Prevention of Inflammatory Bowel Disease. Toxicol Rep (2018) 5:314–7. doi: 10.1016/j.toxrep.2018.02.007 PMC5977373 29854599

[155] Park J-S Choi JW Jhun J Kwon JY Lee B-I Yang CW. Lactobacillus Acidophilus Improves Intestinal Inflammation in an Acute Colitis Mouse Model by Regulation of Th17 and Treg Cell Balance and Fibrosis Development. J Med Food (2018) 21(3):215–24. doi: 10.1089/jmf.2017.3990 29336663

[156] Mallon P McKay D Kirk S Gardiner K. Probiotics for Induction of Remission in Ulcerative Colitis. Cochrane Database Syst Rev (2007) 4):Cd005573. doi: 10.1002/14651858.CD005573.pub2 17943867

[157] Rahimi R Nikfar S Rahimi F Elahi B Derakhshani S Vafaie M. A Meta-Analysis on the Efficacy of Probiotics for Maintenance of Remission and Prevention of Clinical and Endoscopic Relapse in Crohn's Disease. Digest Dis Sci (2008) 53(9):2524–31. doi: 10.1007/s10620-007-0171-0 18270836

[158] Jia K Tong X Wang R Song X. The Clinical Effects of Probiotics for Inflammatory Bowel Disease: A Meta-Analysis. Medicine (2018) 97(51):e13792–e. doi: 10.1097/MD.0000000000013792 PMC6319782 30572537

[159] Ganji-Arjenaki M Rafieian-Kopaei M. Probiotics are a Good Choice in Remission of Inflammatory Bowel Diseases: A Meta Analysis and Systematic Review. J Cell Physiol (2018) 233(3):2091–103. doi: 10.1002/jcp.25911 28294322

[160] Zmora N Zilberman-Schapira G Suez J Mor U Dori-Bachash M Bashiardes S. Personalized Gut Mucosal Colonization Resistance to Empiric Probiotics Is Associated With Unique Host and Microbiome Features. Cell (2018) 174(6):1388–405.e21. doi: 10.1016/j.cell.2018.08.041 30193112

[161] Suez J Zmora N Segal E Elinav E. The Pros, Cons, and Many Unknowns of Probiotics. Nat Med (2019) 25(5):716–29. doi: 10.1038/s41591-019-0439-x 31061539

[162] Lerner A Shoenfeld Y Matthias T. Probiotics: If It Does Not Help It Does Not Do Any Harm. Really? Microorgan (2019) 7(4):104. doi: 10.3390/microorganisms7040104 PMC6517882 30979072

[163] Teshale A Belete A Tilahun A Tsehaye H Berhanu H Minwuyelet A. Bacterial Probiotics Their Importances and Limitations: A Review. . J Nutr Health Sci (2017) 4. doi: 10.15744/2393-9060.4.202

